# Whole genome sequencing puts forward hypotheses on metastasis evolution and therapy in colorectal cancer

**DOI:** 10.1038/s41467-018-07041-z

**Published:** 2018-11-14

**Authors:** Naveed Ishaque, Mohammed L. Abba, Christine Hauser, Nitin Patil, Nagarajan Paramasivam, Daniel Huebschmann, Jörg Hendrik Leupold, Gnana Prakash Balasubramanian, Kortine Kleinheinz, Umut H. Toprak, Barbara Hutter, Axel Benner, Anna Shavinskaya, Chan Zhou, Zuguang Gu, Jules Kerssemakers, Alexander Marx, Marcin Moniuszko, Miroslaw Kozlowski, Joanna Reszec, Jacek Niklinski, Jürgen Eils, Matthias Schlesner, Roland Eils, Benedikt Brors, Heike Allgayer

**Affiliations:** 1 Heidelberg Center for Personalized Oncology, DKFZ-HIPO, DKFZ, Im Neuenheimer Feld 580, 69120 Heidelberg, Germany; 2 Division of Applied Bioinformatics, German Cancer Research Center (DKFZ) and National Center for Tumor Diseases (NCT), 69120 Heidelberg, Germany; 3 Division of Theoretical Bioinformatics, German Cancer Research Center (DKFZ), 69120 Heidelberg, Germany; 4 Department of Experimental Surgery-Cancer Metastasis, Medical Faculty Mannheim, Ruprecht Karls University Heidelberg, 69120 Mannheim, Germany; 5 Centre for Biomedicine and Medical Technology Mannheim (CBTM), 68167 Mannheim, Germany; 6 Medical Faculty Heidelberg, Ruprecht Karls University Heidelberg, 69120 Heidelberg, Germany; 7 Department of Biostatistics, German Cancer Research Centre (DKFZ), 69120 Heidelberg, Germany; 8 Institute of Pathology, University Hospital Mannheim (UMM), 68167 Mannheim, Germany; 9 Faculty of Medicine, Medical University of Bialystok, 15-269 Bialystok, Poland; 10 Department of Bioinformatics and Omics Data Analytics, German Cancer Research Center (DKFZ), 69120 Heidelberg, Germany; 11 Department for Bioinformatics and Functional Genomics, Institute for Pharmacy and Molecular Biotechnology (IPMB) and BioQuant, Ruprecht Karls University Heidelberg, 69120 Heidelberg, Germany; 12 German Cancer Consortium (DKTK), 69120 Heidelberg, Germany

## Abstract

Incomplete understanding of the metastatic process hinders personalized therapy. Here we report the most comprehensive whole-genome study of colorectal metastases vs. matched primary tumors. 65% of somatic mutations originate from a common progenitor, with 15% being tumor- and 19% metastasis-specific, implicating a higher mutation rate in metastases. Tumor- and metastasis-specific mutations harbor elevated levels of BRCAness. We confirm multistage progression with new components *ARHGEF7/ARHGEF33*. Recurrently mutated non-coding elements include ncRNAs *RP11-594N15.3, AC010091, SNHG14*, 3’ UTRs of *FOXP2, DACH2, TRPM3, XKR4, ANO5, CBL, CBLB*, the latter four potentially dual protagonists in metastasis and efferocytosis-/*PD-L1* mediated immunosuppression. Actionable metastasis-specific lesions include *FAT1, FGF1, BRCA2, KDR*, and *AKT2*-, *AKT3*-, and *PDGFRA*-3’ UTRs. Metastasis specific mutations are enriched in PI3K-Akt signaling, cell adhesion, ECM and hepatic stellate activation genes, suggesting genetic programs for site-specific colonization. Our results put forward hypotheses on tumor and metastasis evolution, and evidence for metastasis-specific events relevant for personalized therapy.

## Abstract (web summary)

The evolution and genetic nature of metastatic lesions is not completely characterized. Here the authors perform a comprehensive whole-genome study of colorectal metastases in comparison to matched primary tumors and define a multistage progression model and metastasis-specific changes that, in part, are therapeutically actionable.

## Introduction

Metastasis is the leading cause of cancer-related mortality and remains challenging due to its resistance to therapy, aggressive phenotype and multi-organ affectation^1,2^. Clearly, metastasized lesions behave differently from their precursor primaries and this recognition has led to advancements of several hypotheses, including that of cancer stem-cells to explain this behavior^3^. Accordingly, attempts have been made to identify genetic alterations that differentiate metastatic from primary tumors^4^. Interestingly, most molecular comparisons have been made between advanced primary tumors and early-stage (non-metastasized) tumors, without looking at the metastatic lesions themselves^5,6^. Very few studies have analyzed metastatic lesions with their corresponding primaries; however, these studies were restricted to a defined set of protein coding genes^1,7^. Recent attempts using next generation sequencing have characterized the mutational landscape of solid primary tumors to a greater detail, but done little to add to our knowledge of metastatic disease^4,8–11^. In colorectal cancer, the largest exome studies were by Giannakis et al.^12^ with 619 primary tumor samples, building upon the previous Cancer Genome Atlas (TCGA) study where 276 primary tumors were analyzed^5^. A study by Yaeger et al.^13^ examined 1099 patients using a limited panel of up to 468 genes, but only 18 patients with matched tumor and metastasis samples, while the study by Zie and colleagues looked into both primary colorectal tumors and their metastases, but this study was limited to 2 samples^14^.

Here we present the most comprehensive analysis of whole-genome differences between metastatic lesions and their corresponding primaries in micro satellite stable colorectal cancer samples from patients without a prior familial history of the disease, thus reducing many hidden germline components. Using whole-genome sequencing, we characterize the metastatic lesions of 12 patients (details in Methods, Tables 1 and 2, Supplementary Data 1), together with their primary tumors and corresponding normal samples, assess somatic genomic lesions and mutational signatures, and ascertain similarities, as well as differences between primary tumors and metastases. Although we identify a number of additional non-coding facets of disease progression, more importantly, we assess and identify metastasis-specific clinically relevant mutations and mutational signatures that may impact future therapy decisions. The results put forward novel hypotheses on metastasis evolution and suggest new components of disease progression.

**Table 1:** Patient clinical and sample information

Patient IDs	Age at Surgery	Gender	Diagnosis	Histology	pT	pN	M	Metastasis site
CRC-001	63 years 5 months	Male	Cancer of colon	Adenocarcinoma	3	1	1	Liver
CRC-002	58 years 7 months	Female	Cancer of colon	Tubulo-papillary Adenocarcinoma	2	1	1	Liver
CRC-003	65 years 0 months	Male	Cancer of rectum	Adenocarcinoma	3	2	1	Liver
CRC-004	55 years 11 months	Female	Cancer of rectum	Mildly differentiated Adenocarcinoma	3	2	1	Lung
CRC-005	48 years 6 months	Male	Cancer of rectum	Moderately differentiated Adenocarcinoma	4	1	1	Liver
CRC-006	55 years 10 months	Female	Cancer of rectum	Adenocarcinoma	3	2	1	Liver
CRC-007	64 years 7 months	Male	Cancer of rectum/colon	Tubulo-papillary Adenocarcinoma	3	2	1	Liver
CRC-008	48 years 9 months	Male	Cancer of rectum	Adenocarcinoma	3	1	1	Liver
CRC-009	70 years 5 months	Male	Cancer of colon	Tubulo-papillary Adenocarcinoma	4	2	1	Liver
CRC-010	68 years 1 months	Female	Cancer of colon	Moderately differentiated Adenocarcinoma	3	2	1	Liver
CRC-011	59 years 9 months	Male	Cancer of rectum	Adenocarcinoma	3	1	1	Liver
CRC-012	62 years 9 months	Male	Cancer of rectum	Tubulo-papillary Adenocarcinoma	3	0	1	Liver

Table displaying the anonymized/pseudonymized patient ID, age, gender, diagnosis and histology of patients/tumors. The initial staging of the disease is shown in fields for primary tumor (pT), regional lymph nodes (pN), distant metastasis (M)

**Table 2:** Patient clinical and sample information, continued

Tumor location (site)	Pre-surgical therapy	Tumor cell content (ACEseq)	Tumor ploidy (ACEseq)	Metastasis cell content (ACEseq)	Metastasis ploidy (ACEseq)
Sigmoid colon (left)	-	0.85	2.16	0.57	2.28
Transverse colon (right)	-	0.55	3.28	0.56	3.33
Rectum (left)	Neo-adjuvant RCTX	0.6	3.44	below 0.3	N/A
Rectum (left)	-	0.69	3.12	0.67	3.03
Rectum (left)	-	0.39	3.08	0.4	2.75
Rectum (left)	Neo-adjuvant RCTX	0.36	2.19	0.63	2.21
Recto sigmoid (left)	-	0.61	3.49	0.48	3.41
Rectum (left)	-	0.42	3.72	0.31	3.73
Sigmoid colon (left)	-	below 0.3	N/A	below 0.3	N/A
Caecum (right)	-	0.68	2	0.65	1.73
Rectum (left)	-	0.49	3.87	0.37	3.9
Rectum (left)	Neo-adjuvant RCTX	below 0.3	N/A	0.86	2.28

Tumor site and location, pre-surgical therapy, tumor cell content, tumor ploidy, metastasis cell content and metastasis ploidy are listed. RCTX abbreviates radio-chemo therapy

## Results

### Somatic single nucleotide variations, mutations, and indels

First, we determined the mutational load in the 12 resected metastasis samples as compared to the matched primaries (Supplementary Data 2, Supplementary Figure 1). We observed a median of 10,468 (range 5773–16,934) and 11,475 (range 4774–17,189) somatic single nucleotide variations (SNVs) in the tumor and metastasis samples with high tumor cellularity, consistent with our samples being non-hyper mutated, non-ultra-mutated and microsatellite stable (MSS). We found that 65% (36–92%) of all SNVs were shared between tumors and corresponding metastases, clearly suggesting a common ancestral truncal clone with 15% (1–29%) tumor-specific and 19% metastasis-specific (3–42%), respectively (Supplementary Data 2). This suggests that the rate of mutation is higher in the metastatic lesion compared to the matched tumor after truncal separation.

**Fig. 1:**
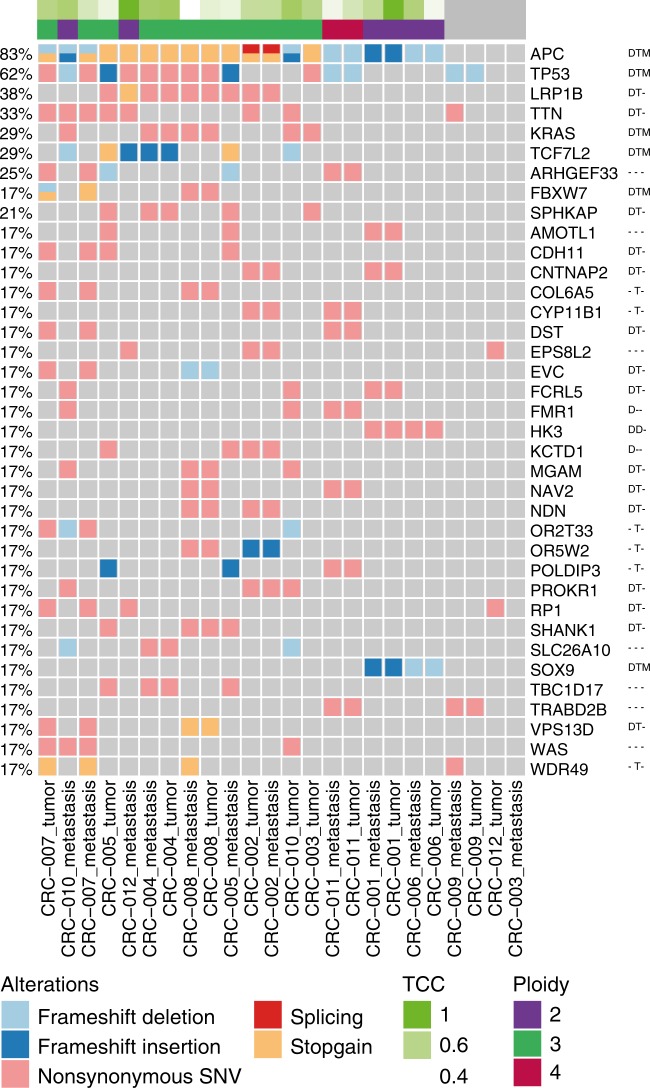
Recurrent somatic small mutations on protein coding genes. Oncoprint representation of recurrently mutated genes with a cutoff of 4 samples (17%). The top annotation shows the tumor cell content (TCC) and estimated tumor ploidy. The color of the box indicates the type of mutation. Recurrently mutated genes are marked with D, T, M if they were also recurrently mutated (>2.5%) in the Giannakis et al./DFCI 2016 (D), TCGA provision (T) and Yaeger et al./MSK-CC 2018 cohorts (M) via cBioPortal

Next, we investigated recurrent coding mutations. The most recurrently mutated genes are well established in colorectal carcinogenesis (Fig. 1). Mutations in these driver genes were present in both tumor and metastasis high-purity pairs, apart from colorectal cancer (CRC) patient CRC-010 where the *TP53* mutation was only observed in the metastasis sample. In addition to these, we observed recurrent mutations in *ARHGEF33*, a guanine nucleotide exchange factor (GNEF) that facilitates small GTPases like *KRAS*, and *SPHKAP*, which encodes an A-kinase anchor protein.

Furthermore, we also found previously undescribed recurrently mutated non-protein coding genes in tumors and metastases (Supplementary Figure 2, Supplementary Data 3). These included *AC010091.1*, *CTD-2292P10.4*, *RP11-594N15.3*, and *SNHG14*. *AC010091.1* shares homology with protocadherin *FAT4*, which negatively regulates Wnt signaling and its knockdown induces epithelial–mesenchymal transition (EMT) in gastric cancer (GC)^15^. *SNHG14* has been shown to bind directly to miR-145-5p^16^, a potent tumor suppressor in multiple cancer types^17^. The non-coding ribonucleic acid (ncRNA) *RP11-421L10.1* was more recurrently mutated in metastasis (3 vs 1).

In 3′-untranslated regions (UTRs), commonly affected genes included *XKR4*, *ANO5*, *FOXP2*, *CBL*, *CBLB*, *NTRK3*, *TRPM3*, *DACH2*, the latter 2 also more recurrently mutated in metastases (3 vs 2) and *FOXP2* only in diploids (Supplementary Figure 2). We observed that 3′-UTR mutations of *XKR4* were mutually exclusive to *ANO5* (i.e., patients with *XKR4* 3′-UTR mutations did not harbor *ANO5* 3′-UTR mutations, or vice versa). These genes are paralogs of *XKR8* and *ANO6*/*TMEM16F*, which mediate an externalization of phosphatidyl serine, creating an immunosuppressive tumor micro environment^18^. Likewise, samples with mutations in the 3′-UTR of E3 ubiquitin-protein ligase *CBL* showed mutual exclusivity to its paralogue *CBLB*. These genes have been shown to inhibit EGFR signaling through degrading EGFR and binding to GRB2^19^. CBL has also been described to be involved in cancer progression and metastasis^20^, the nuclear degradation of β-catenin^21^, and to downregulate *PD-L1* in non-small cell lung cancer^22^. FOXP2 has also been shown to bind to and downregulate *CNTNAP2*^23^. We evaluated potential perturbations in miRNA mediated messenger RNA (mRNA) stability caused by these 3′-UTR mutations in silico (Supplementary Data 4). In patient CRC-006, a mutation in the 3′-UTR of *FOXP2* causes the potential loss of regulation by miR-670-5p, miR-3912-5p, miR-4669, miR-6753-3p, and miR-190b, which has been shown to bind to the *FOXP2* 3′-UTR in gastric cancer (GC)^24^. In CRC-004, a mutation causes the targeting of the *XKR4* 3′-UTR by 7 additional miRNAs and in CRC-007, a mutation in the 3′-UTR of the same gene results in enhanced interaction of miR-1293. Similarly, in CRC-011, a mutation in the 3′-UTR of *ANO5* causes a loss of binding for 6 miRNAs; however, binding is enhanced for 13 additional miRNAs shifting the flux towards mRNA degradation.

### Copy number aberrations

**Fig. 2:**
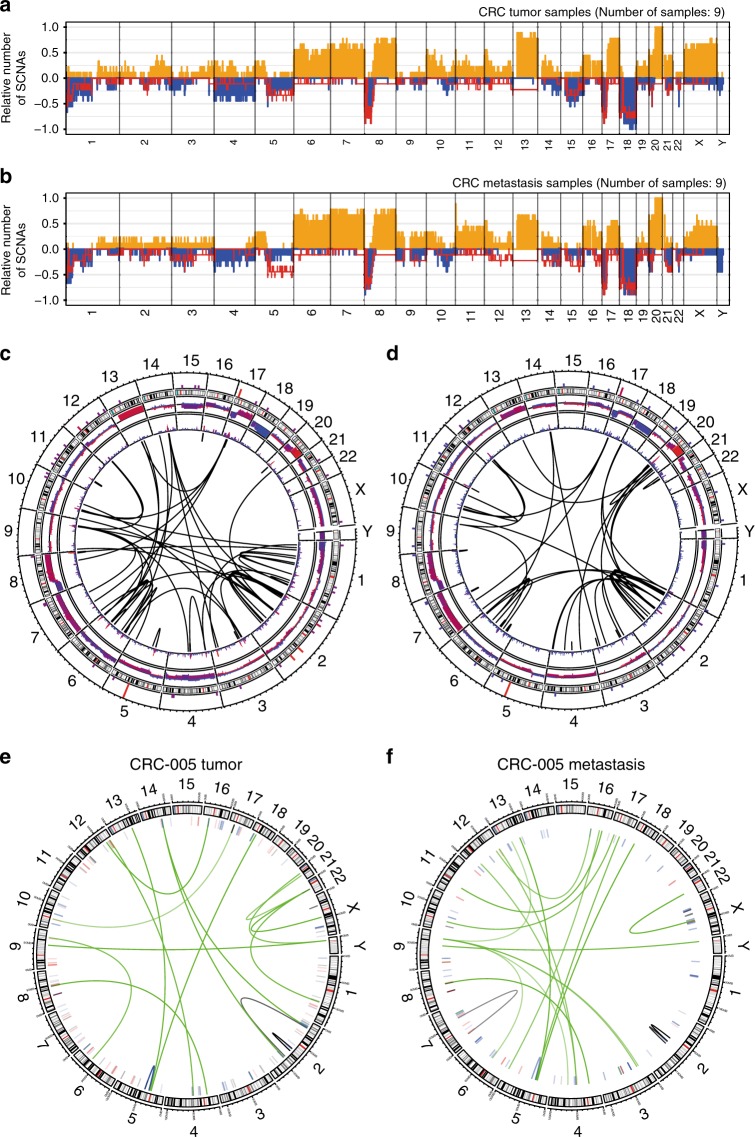
Recurrent somatic copy number aberrations and structural variations. Relative prevalence of somatic copy number aberrations (predicted by ACEseq) in high tumor cell content (TCC) primary tumors **a** and metastasis **b** samples, showing presence of at least one copy number gain (orange bars), copy number loss (blue bars), and LOH (red line) as a proportion of analyzed samples. Circular plots of recurrent (minimum of 3) somatic structural variations (SVs) in high TCC tumors **c** and metastasis **d** samples. The panels (from outside going inwards) represent small somatic variant recurrence per gene, genomic cytobands, copy number changes (predicted by SOPHIA), and recurrent SVs within TAD regions. As an example of SV heterogeneity, we show the SV landscape for CRC-005 tumor **e** and metastasis **f**. Arcs represent translocation and inversion events

Copy number aberration (CNA) patterns were similar in tumors and metastases (Fig. 2, Supplementary Figure 3). In addition to recurrent arm level events found in the TCGA study^5^, we observed recurrent amplifications of chromosome arms 6p and q and 16p and losses in 4p, 5q, 8p. The gains on chromosome 4 seen in tumors were virtually absent in metastases (Fig. 2a, b). Further differences include gains of chromosomes 9, 11 and loss of Y, which were more frequent in metastasis samples, and gains of chromosomes 2q, 10p, 13, 17, 21 and X and losses of 15 which were less frequent.

**Fig. 3:**
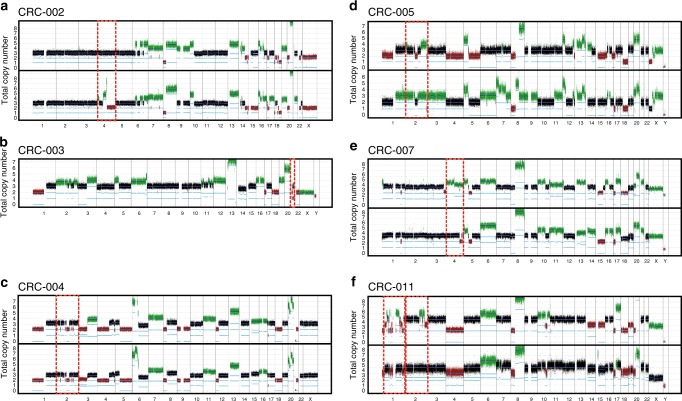
Chromothripsis and negative selection of highly rearranged chromosomes. Chromosome copy number predictions of six samples **a**–**f**, showing predicted copy number of tumor (top) and metastasis (bottom) samples. Regions of chromothripsis-like rearrangements **b**, **c**, **f** and highly rearranged events not present in the metastasis **a**, **d**, **e**, **f** are highlighted in dashed red boxes

We also observed chromothripsis-like chromosomal rearrangements in five samples, all of which carried a *TP53* mutation. Certain high level genomic rearrangements did not persist in the metastasis (Fig. 3).

We also compared copy number aberrations with miRNA gene expression changes^25^, and found amplifications associated with the increased expression of *miR-483*, *miR-409*, *miR-411*, *miR-134*, *miR-154*, *miR-654*, *miR-299*, *miR-382*, *miR-379*, and *miR-487b* in the metastases. Deletions coupled with reduced expression were observed for *miR-34a*, *miR-552*, *miR-30e*, and *miR-122* in primaries or metastases.

### Structural variations

*MACROD2* was the gene most recurrently hit by structural variations (SVs), followed by *PDE11A*, *TTC28*, *FHIT*, and *PARK2* (Fig. 3, Supplementary Figure 4, Supplementary Figure 5, Supplementary Data 5). *MACROD2*^26^, *FHIT*, and *PARK2* are located on chromosomal fragile sites and their deletions are indicative of replication stress. Remarkably, one of the most frequently deleted loci in the TCGA study, *RBFOX1*, did not show frequent events in our cohort. The few cases (4 of 12) where *RBFOX1* showed deletions were tumor-specific events, suggesting negative selection of *RBFOX1* in metastasis. Structural aberrations involving *SAMD5*, *MACROD2*, *IGF2* and the non-coding gene *AC007319.1* were found to be more recurrent in metastasis. SVs involving *ARHGEF18*, *IFNGR2*, *RBFOX1*, *SLIT3*, *TMEM50B*, non-coding genes *CTD-2374C24.1*, *RP11-6N13.1*, *RP11-420N3.2*, *CTD-2207O23*.3, and *CTC-575N7*.1 were seen more recurrently in primary tumors (Supplementary Data 5).

### An extended colorectal cancer progression model

**Fig. 4:**
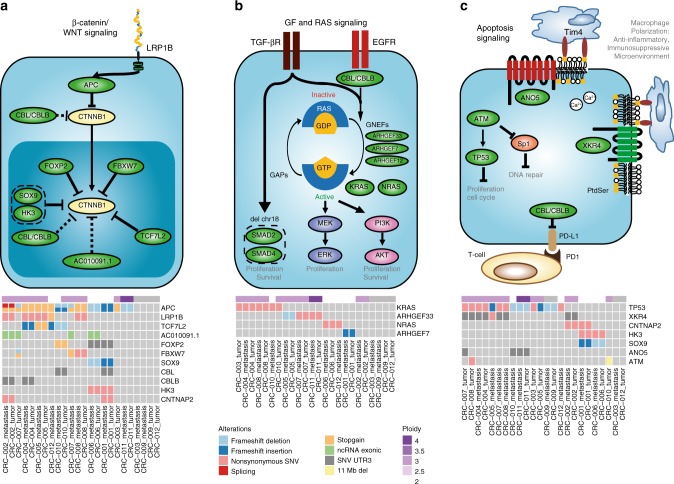
Pathway model of colorectal cancer molecular drivers. Cartoon model (top) and oncoprints (bottom) of somatically mutated genes within colorectal progression pathways. Models and oncoprints of recurrently mutated genes within β-catenin/Wnt **a**, growth factor & RAS **b**, and apoptosis signaling **c** pathways. Genes were identified based on mutual exclusivity analysis and literature. Genes identified to be mutated in this study are shown in green ovals

The classical model of colorectal cancer progression^27^ describes sequential gains of mutations in Wnt signaling, RAS signaling, TGF-beta signaling and p53 signaling. Performing mutual exclusivity and co-occurrence analysis allowed us to place additional components to this model (Fig. 4, Supplementary Data 6). We identified highly redundant mutational targeting of negative regulators of the Wnt signaling (Fig. 4a), with 85% of high-purity samples having mutations in 3 recurrently mutated regulators. Although we confirm known regulators, including *APC*, *TCF7L2*, *FBXW7*, and *SOX9* (of which the latter 3 are mutually exclusive), we show that *SOX9* is mutated in diploid only samples. This is further supported by a significant mutual exclusivity of *SOX9* mutations with *TP53* mutations (associated with aneuploidy) in the TCGA cohort (*p*-value 0.025, Fisher exact test). *AC010091.1*, mutated in 25% of samples, may play a role in the nuclear regulation of β-catenin as a decoy for miRNAs targeting *FAT4*, a suppressor of Wnt signaling^28^. Mutations in *AC010091.1* were mutually exclusive to *TCF7L2* and *KRAS*. Our data also suggest that *LRP1B*, a negative regulator of Wnt signaling that is downregulated in right-sided colorectal cancer (rCRC)^29^, may play a role in Wnt signaling upstream of APC, as an alternative to the TCGA’s proposed *LRP5*. *LRP1B* mutations were nearly always associated with triploidy. Mutual exclusivity of 3′-UTR mutations in *CBL* and *CBLB* implicate them as regulators of tumorigenic β-catenin^21^ independent of *FBXW7*. However, *CBL* and *CBLB* may play a dual role, as they have also been implicated in downregulation of EGFR signaling.

We observed mutual exclusivity of *KRAS*, *NRAS* mutations and guanine nucleotide exchange factors *ARHGEF33* and *ARHGEF7* (Fig. 4b), suggesting that these may play a similar role to *KRAS* and *NRAS* mutations. Other studies also showed recurrent mutations in *ARHGEF* genes (Supplementary Figure 6) and the distribution of mutations in several *ARHGEF* genes clustered toward the RhoGEF and Plekstrin homology (PH) domains (Supplementary Figure 7). In the TCGA series, we find that *ARHGEF7* mutations associate with worse disease-free survival (*p*-value 0.004, logrank test) and generally, patients with *ARHGEF* mutations show worse disease-free survival (*p*-value 0.04) (Supplementary Figure 8). In our present series, *NRAS* and *ARHGEF7* were mutated only in diploid samples, while *KRAS* and *ARHGEF33* mutations were associated with aneuploidy and *TP53* mutations in all but 1 case.

We did not observe recurrent small mutations on components of TGF-β signaling; however, all but one of our samples exhibited loss of chromosome 18 which contains the key genes *SMAD2* and *SMAD4* (Fig. 4b). This loss of chr18 has also been associated with hepatic metastasis^30^.

**Fig. 5:**
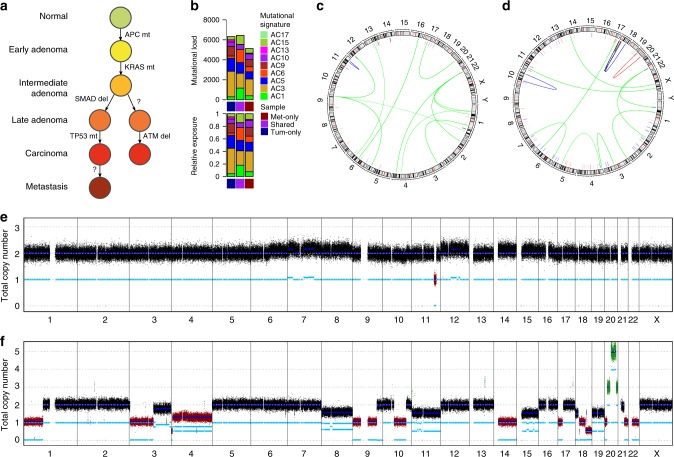
Genomic landscape of tumor and metastasis mutations in patient CRC-010. Model of progression of tumor and metastasis from normal epithelial cells **a**, mutational signatures for tumor (dark blue), shared (red), and metastasis (dark red)-specific mutations **b**, structural variations in tumor **c** and metastasis **d** and copy number profiles in tumor **e** and metastasis **f**. *ATM* is located in the small deleted segment of chromosome 11 in the tumor sample **d**

Mutations in *TP53* were associated with aneuploidy (Fig. 4c). Although most *TP53* mutations were present in both tumor and metastasis samples, CRC-010 exhibited a *TP53* mutation in the metastasis, but not the primary tumor which instead had an 11 Mb deletion spanning *ATM*, a regulator of TP53 (Fig. 5). This suggested two independent carcinoma triggering events in this patient. In line with evasion of apoptosis, we propose a potential role of perturbed phosphatidyl serine externalization facilitating immune evasion, by dysregulating 3′-UTR mutations of the *XKR* and *TMEM16* family genes *XKR4* (exclusively mutated in triploids) and *ANO5* (*TMEM16E*). Recently, it has been shown that both *CBL* and *CBLB* play a role in modulating expression of programmed death ligand 1 (*PD-L1*), thus also playing a role in immune evasion (Fig. 4c).

**Fig. 6:**
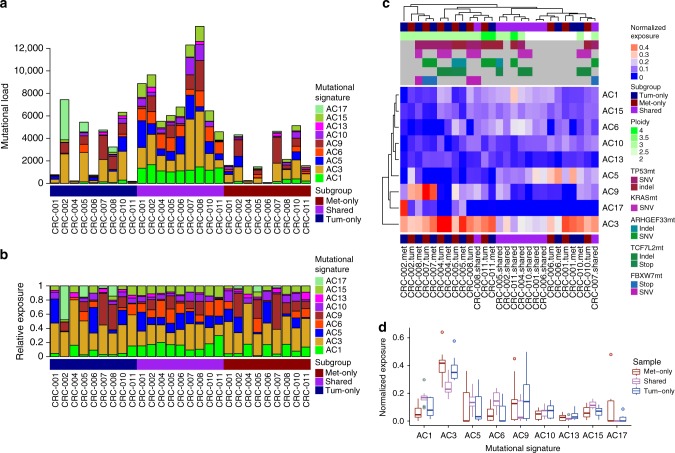
Mutational signatures in colorectal cancer progression. Bar plot representation of absolute **a**, and normalized **b** COSMIC cancer mutational signatures within the strata of tumor-specific (dark blue), metastasis-specific (dark red) and shared (purple) somatic SNVs per patient with high tumor cell content (TCC). Unsupervised clustering of normalized exposures, with top annotation showing ploidy, estimated TCC and mutational status for *TP53, KRAS, ARHGEF33, TCF7L2*, and *FBXW7* **c**. Box and whisker plot of distributions of normalized exposures between mutations that are tumor-specific (dark blue), metastasis-specific (dark red), and shared (purple) per patient **d**. Boxes denote the interquartile range, the middle line denotes the median, and the vertical lines outside the box denote the minimal and maximum range excluding outliers (which are 1.5 times the interquartile range)

Finally, by stratification of the mutational catalog, we were able to identify signatures particular to early-stage development (Fig. 6), and later evolution of the resultant tumor and metastasis samples. We observed more prominent DNA mismatch repair (MMR) defect signatures (AC6 and AC15) in early development, which seemed to be replaced by gain of a DNA, double-strand break-repair by homologous recombination (DSB) repair defective signature (AC3) in later stages (see following section).

### Mutational patterns and signatures in disease progression

We sought to identify additional patterns that would potentially be indicative of disease progression after finding evidence for an increased mutational rate in metastases as compared to primaries. Looking into cancer mutational signatures^31^ of the stratified catalog of tumor-specific, metastasis-specific, and shared mutations, we found signatures AC1, AC3, AC5, AC6 and AC9, AC10, AC13, AC15 and AC17 (Fig. 6a, b). Signatures AC1 and AC5 are believed to be caused by age-related clock-like mutagenic processes, AC1 initiated by spontaneous deamination and AC5 by an unknown mechanism. Signatures AC3, AC6, and AC15 have been associated with failure of DNA repair systems, in case of AC3 by failure of double-strand break-repair by homologous recombination and in case of AC6 and AC15 by failure of mismatch repair (MMR); signature AC9 is attributed to the activity of activation-induced (Cytidine) deaminase (AID). Signature AC10 has been linked to altered polymerase (POL) E function, signature AC13 is linked to the activity of members of the APOBEC enzyme family and signature AC17 has not been associated with a specific mechanism yet.

Unsupervised clustering of the stratified catalogs based on normalized exposures of mutational signatures revealed a significant association between ploidy and the clock-like signatures AC1 and AC5: high exposure to AC1 is associated with polyploidy, whereas enrichment of AC5 is associated with diploidy (Fig. 6c).

Comparing normalized exposures in different strata of SNVs, the clock-like signature AC1 (spontaneous deamination) is more truncal (significant before, trend after Benjamini-Hochberg (BH)-correction). Furthermore, we observed differences in DNA repair defect signatures: AC6 and AC15 (MMR) are truncal (significant before, trend after BH-correction), whereas AC3 (DSB, BRCA-ness) is an ongoing mutational process with significantly higher contributions in the strata private to tumors and metastases (Fig. 6d). This again supports the hypothesis of a common ancestor clone between tumor and metastasis with altered late stage mutagenic processes ongoing after truncal separation.

### Functional relevance of metastasis-specific mutations

We found 48 genes to be mutated in metastases but less so in primary tumors. Performing functional annotation clustering analysis, we found extracellular matrix, PI3K-Akt signaling, and focal adhesion-related pathways to be significantly enriched in metastases (*p*-value of 1.2 × 10^−11^, 2.7 × 10^−10^, and 2.2 × 10^−5^, respectively; BH corrected hypergeometric test; Supplementary Figure 9, Supplementary Data 7). Of these 48 genes, 12 were present in the matrisome of metastatic CRC tumor samples of which 11 had lower protein abundance in the metastasis samples^32^, including ADAMTSL1, which was a colon tumor-specific extracellular matrix (ECM) protein, not present in normal colon, metastasis nor liver tissue. None of the metastasis-specific ECM proteins were found in the list of 48 mutated genes.

Additionally, looking at canonical pathways enriched either in tumor or metastasis specifically mutated genes, we found that hepatic fibrosis/stellate cell and actin cytoskeleton cascades were significantly enriched in metastasis (Supplementary Figure 9). As almost all our sequenced metastatic lesions were in the liver, it appears that metastasized cells invoke a response that in some way fosters organ-specific metastatic colonization.

### Clinical relevance of metastasis-specific mutations

Genomic alterations in the metastasis genome are clinically relevant if they are actionable (for therapy or decision-making) and more so if they differ from that of the primary tumor. To analytically evaluate such alterations, we used the TARGET database as well as the database of the NCT-MASTER program^33^ to ascertain potentially clinically relevant events in the tumor and metastasis tissues for individual patients. The number of these mutations in the individual patients ranged from 1 to 17, with an average of nine mutations per sample. Most clinically relevant mutations were identical between tumor and metastasis samples from the same patients. However, in four patients, we found clinically relevant metastasis-specific non-silent mutations of *FAT1*, *FGF1*, *BRCA2*, *TP53*, and *KDR* and tumor-specific splice site mutations of *JAK2* (Supplementary Data 8). We also searched for alterations in the 3′-UTRs of potentially targetable genes and discovered, with the exception of two patients, at least one per patient affecting different genes. Interestingly, three patients harbored 3′-UTR mutations in genes of clinical interest: *AKT3* (CRC-002), *PDGFRA* (CRC-005), and *AKT2* (CRC-010) (Supplementary Data 9).

We also observed *EGFR* amplifications in the metastasis sample of CRC-005 (4 copies) compared to the tumor (3 copies), implicating consequences for EGFR-based targeted therapy of certain metastases.

**Fig. 7:**
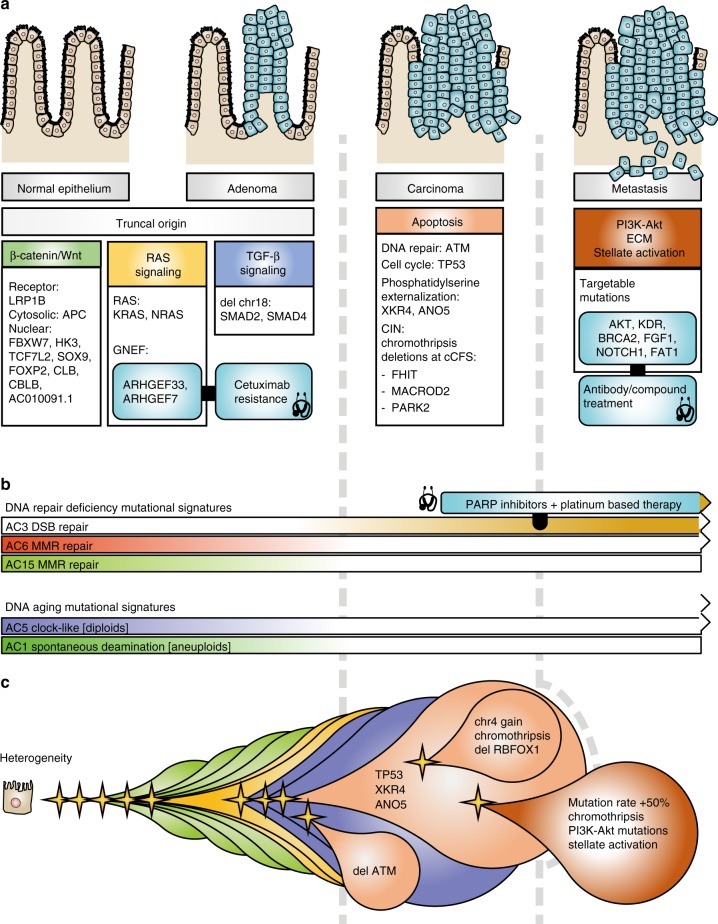
Model of colorectal cancer and metastasis progression and therapeutic implications. A summary cartoon of how recurrent somatic mutations identified within this study fit into established colorectal progression models **a**. The top cartoons represent the transition from normal epithelial cells to metastasis (left to right). Beneath the cartoons are tables of genes and genetic lesions that were mutated in our cohort, sorted in tables related to possible pathway function. Change of relative exposure to mutational signatures are shown as horizontal bars where the strength of exposure corresponds to the strength of color in the bar, relative to tumor evolution (left to right) **b**. Cartoon representation of lesion (stars) accumulation giving rise to tumor heterogeneity **c**. Balloons are colored according to pathways, as the table headers in **a**, showing mutations in Wnt (green), RAS (orange), TGF-β (blue) signaling, and mutations acquired in carcinogenesis (brown), and metastasis (dark brown) formation. We show events which might not give rise to further progression. Mutations with implications on therapy decision are shown in light blue boxes with rounded corners, and linked to boxes with therapy consideration via a thick black line **a**, **b**. Gray vertical dashed lines separate out lesions corresponding to truncal origin, tumor, and metastasis states

Finally, we observed a significantly reduced defective DNA mismatch repair signature (AC3) in the tumor and metastasis-specific mutations compared to the truncal node, but persistence of BRCA-ness mutational signatures, suggesting possible efficacy of PARP inhibitor treatment for both the primary tumors and metastases. An overview of our findings and suggestion of an extended progression model of colorectal cancer and its metastasis is shown in Fig. 7.

## Discussion

This is the most comprehensive study to date systematically describing whole-genome landscape differences in tumor and metastatic lesions of colorectal cancer. In our study, an average of 65% of all somatic SNVs were shared between tumors and corresponding metastases; an average of 15% were specific to tumors and an average of 19% specific for metastases, suggesting that the rate of mutagenesis is higher in the metastatic clone compared to the primary tumor.

In line with the Vogelstein model^27^, we revealed additional protein coding and non-coding components and implicate dependency of existing mechanisms to ploidy state. Thus, the model can now be further refined^34^. The initial lesion for non-hyper mutated/microsatellite stable tumors is adenoma genesis via redundant perturbations in Wnt signaling leading to over expression of β-catenin for which we identified components *LRP1B*, *AC010091.2*, *CBL*, and *CBLB*. We hypothesize that the guanine nucleotide exchange factors *ARHGEF33* and *ARHGEF7* may play a similar role to *KRAS* and *NRAS* mutations. While these *ARHGEF* genes were identified in our series, we found a number of other *ARHGEFs* that exhibited clustered and recurrent mutations on functional domains, further implicating an important and yet unexplored role of *ARHGEF* genes. This is of special importance as patients with *KRAS* and *NRAS* mutations do not respond well to EGFR inhibitors panitumumab and cetuximab^35^, which may mean that patient CRC-005 that exhibited an *EGFR* amplification in the metastasis but also carried an *ARHGEF33* mutation, may not respond to EGFR inhibitor therapy. While the role in TP53 in carcinoma formation is well known, we postulate the role of perturbed phosphatidyl serine externalization interfering with efferocytosis as a result of potential dysregulation of *XKR4* and *ANO5* by 3′-UTR mutations, which we believe work co-operatively with *TP53* mutations.

The clock-like signature AC1, scaling with the number of passed cell cycles^36^, is enriched in polyploid samples, whereas signature AC5, scaling with elapsed time, is enriched in the diploid samples. A possible interpretation is that rapidly cycling tumors are more prone to be associated with gross karyotypic abnormalities. This could stratify patients into clinical subgroups of better responders to drugs with strong anti-proliferative activity, such as 5-fluorouracil (5-FU). Defective DNA DSB repair machinery as indicated by mutational signature AC3 can be targeted by PARP inhibitors. PARP inhibitors could be used not only as chemo/radiotherapy sensitizers, but as single agents to selectively kill cancers defective in DNA DSB repair while overcoming typical resistance of MMR defective tumors to chemotherapy^37^.

The clinically relevant genes that we found which were exclusive to metastatic lesions include *FAT atypical cadherin 1* (*FAT1*), which regulates cell adhesion, migration, EMT and stemness properties. Somatic mutations of *FAT1* have been found to lead to aberrant Wnt activation in multiple human cancers^38^. *FAT1* is widely expressed in metastatic CRC and can be targeted directly with monoclonal antibody mAb198.3^39^. Fibroblast growth factor 1 (FGF1) is targetable indirectly through its receptors, FGFRs, with agents, including Nintedanib, Pazopanib, Ponatinib^40^. The Kinase insert domain receptor (KDR/VEGFR), functions as the main mediator of VEGF-induced proliferation, survival, migration, and sprouting, and is amenable to drugs, including axitinib, sorafenib, and cabozantinib^41^.

We identified recurrent chromosome arm level events and highlight differences between tumor and metastasis samples. There is an evidence that loss of chromosome 4 is associated with lymph node metastasis, metastatic recurrence, and early micrometastasis^42,43^. Similar reporting of chromosome 4 amplifications in primary tumors but not their matched metastases has been described for metastatic melanomas^44^, which was localized to 4q12-q13.1 which includes *PDGFRA*, *KIT*, *KDR*, and *REST*. *PDGFRA* and *KDR* are important for gain of metastatic potential by driving EMT and proliferation^45,46^. KIT and REST have been implicated as tumor and metastasis suppressors in colorectal cancer^47,48^. Perhaps, this schizophrenic region drives heterogeneity where amplification increases proliferation while reducing its metastatic potential, whereas deletions lead to lower levels of KIT and REST, thus more viable to metastasize.

Importantly, facilitated by whole-genome sequencing, non-coding genes and 3′-UTRs provided significant contributions to the better known protein-coding mutational landscapes of CRCs^49–51^. At present, it is difficult to completely appreciate their impact as their functions are still poorly understood. Certainly, in an earlier publication, we have described the metastasis-specific microRNA landscape and many of the genomic changes are able to offer putative explanations for particular miRs we have described to be deregulated in expression in metastasis^25^. We observed metastasis-specific 3′-UTR mutations in *AKT2* and *AKT3*. Furthermore, in our study, there is an indication of the importance of 3′-UTR mutations in *CBL* and *CBLB*, which plays multiple roles including degradation of tumorigenic β-catenin (encoded by the *CTNNB1* gene) in colorectal cancer^21^, degradation of EGFR^19^, and suppressing the expression of *PD-L1*^22^. We also observed that the most frequent 3′-UTR mutation, which occurred in *XKR4,* was exclusively in triploid samples, and exhibited mutual exclusivity to 3′-UTR mutations in *ANO5*, potentially interfering with efferocytosis. Mutations for both these genes facilitated binding of additional miRNAs in silico^18^. Together, the potential combined effect of modulation of macrophages via efferocytosis and T-cells via *PD-L1* expression, prime a favorable tumor-microenvironment raising the importance of dysregulation of *CBL*, *CBLB*, *XKR4* and *ANO5* in colorectal carcinoma.

Another highlight is the finding that metastatic lesions are enriched in mutations of genes affecting PI3K-Akt signaling, cell adhesion, extracellular matrix, and stellate-cell activation in the liver, the predominant metastasis site in our patient, which we hypothesize is critical for homing within the metastatic niche. This supports the notion that sporadic genetic changes are priming metastatic colonization of tumors to a specific metastatic site, and this is perhaps where the fundamental differences between tumors and metastases lie. Extensive investigations are needed to evaluate functionality of these hypotheses.

Taken together, metastases and tumor genome landscapes are very similar, but definitely not identical, which supports the hypothesis of a divergent evolution of metastatic lesions as compared to the primary tumor after truncal separation. While most of our samples support a late dissemination model, the independent carcinoma triggering events in patient CRC-010 would argue for an early metastasis model^52^, with the split occurring after the intermediate adenoma. In individual cases, actionable mutations private to metastatic lesions are evident. This clearly may warrant clinical consequences and a re-structuration of current personalized therapy concepts aiming at metastasis prevention.

## Methods

### Patient material

Primary tumor, matched metastases and corresponding normal tissues of 12 patients with colorectal cancer were obtained at the Medical Faculty Mannheim, University of Heidelberg, Germany (Tables 1 and 2). The tissue banking and sample study was approved by the Ethical Committee of the University Hospital Mannheim, Medical Faculty Mannheim of Heidelberg University, all relevant ethical regulations were complied with, and informed consent was obtained from all patients or their spouses/relatives when the former were deceased. Bio banking and handling of the tissues followed the BRISQ guidelines^53^.

### Genomic DNA isolation

Genomic DNA was isolated from 5 to 10, 20 μM cryosection slices (depending on tissue size) using the QIAamp DNA mini kit (Qiagen, Hilden, Germany) according to the manufacturer’s manual. The extracted DNA was submitted to the HIPO Sample Processing Laboratory (HIPO-SPL) for quality check and pseudo-anonymization of the samples, then transferred to the Genomics and Proteomics Core Facility of the German Cancer Research Center for sequencing.

### Whole-genome sequencing and alignment

Whole-genome DNA sequencing was performed on the HiSeq2000 platform. Library preparation and whole-genome sequencing of matched tumor/normal/metastasis DNA was carried out^54^. Briefly, 1–5 μg of genomic DNA was fragmented to ~300 bp and size selection conducted by agarose gel excision. Sequencing reads were mapped and aligned using the DKFZ alignment workflow from ICGC Pan-Cancer Analysis of Whole Genome projects [https://dockstore.org/containers/quay.io/pancancer/pcawg-bwa-mem-workflow]. Read pairs were mapped to the 1000 Genomes Project phase 2 assembly of the human reference genome (hs37d5) using Burrows-Wheeler Aligner software^55^ (version 0.6.2) using default parameters apart from -T 0. Duplicates were marked with biobambam (version 0.0.148). Single nucleotide variants and indels (insertion or deletion) of the most significant findings were validated by polymerase chain reaction (PCR) using primers that flanked the mutated sequence. Sanger sequencing was done followed by comparisons to the germline genome sequence for confirmation.

### Small variant calling

Small variants were called from the whole aligned whole-genome sequencing data. They were initially called using our in-house workflows, described below, followed by cross checking of variant positions between tumor and metastasis pairs. SNVs were initially called using the DKFZ SNV and indel calling workflow from ICGC Pan-Cancer Analysis of Whole Genome projects [https://dockstore.org/containers/quay.io/pancancer/pcawg-dkfz-workflow]^54,56^. Briefly, the SNVs were called using samtools and bcftools version 0.1.19^57^ determined to be somatic or germline by comparing the tumor/metastasis sample to the control, and later assigned a confidence. The confidence score was initially set to 10, and subsequently reduced based on overlaps with repeats, DAC blacklisted regions, DUKE excluded regions, self-chain regions, segmental duplication records as introduced by the ENCODE project^58^ and additionally if the SNV exhibited PCR or sequencing strand bias. SNVs with confidence lower than 8 were excluded. Annovar (release Feb 2016)^59^ using gene models from Gencode version 19 were used to annotate SNVs.

Due to potential tumor in normal contamination leading to false negative calls we applied the TiNDA (tumor in normal detection algorithm) workflow (unpublished). Briefly, using the unique set of combined mutated positions for a tumor metastasis pair the B-allele frequency (BAF) was calculated from the tumor, metastasis and control samples. Positions overlapping with common variants were filtered out. Then, the clustering algorithm from Canopy^60^ was applied to the BAF values for the positions in tumor/metastasis vs the control using a single pass run, assuming 9 clusters. The clusters that were determined to be tumor-in-normal had to have 75% of positions above the identity line, the tumor/metastasis mutant allele fraction (MAF) above 1% and the control MAF below 45%. These identified mutations were then reclassified as somatic instead of the original germline annotation.

Indels were initially called using Platypus^61^ version 0.8.4. Platypus filters were used to calculate a confidence score ranging from 0 to 10. Indels with confidence lower than 8 were excluded. Annovar was used to annotate indels.

Due to varying tumor cell content, we cross checked allele frequencies of mutations between tumors and metastasis to validate those small mutations were not missed due to lower tumor cell content in either the tumor or metastasis samples. A SNV was called when (i) it was called somatic using our in-house workflow, (ii) it was called somatic in the matched tumor/metastasis pair and its MAF was above 5% (corresponding to a minimum of 2 reads) and at least twice that of the matched germline control. This threshold of 2 reads was selected based on our series (i) where some of the samples are triploid (median series ploidy) (ii) with ×36 coverage (median series coverage), (iii) and with a tumor purity of 47.5% (median series purity), where the expected read support for a single copy variant would be 5.7 reads (0.475 × 36/3). Using a Poisson distribution model, variants with 2 read support fall within the majority of the distributions (pPoisson (*X* = 2) = 0.54, where *μ* = 5.7). SNVs that were shared between tumor and metastasis samples tended to have similar variant allele fractions (VAF) (Supplementary Figure 1). In some samples (CRC-004, CRC-006, and CRC-007) we observed slightly lower VAF in the tumor- and metastasis-specific mutations compared to the shared mutations, indicating a dominant truncal clone with low level heterogeneity.

We classify mutations of interest as somatic SNV and indels those causing protein coding changes (non-silent), and also exonic mutations on non-coding genes. Annotation of non-silent mutations in protein coding genes include non-synonymous SNVs, gain or loss of stop codons, splice site mutations, and both frameshift and non-frameshift indels in protein coding genes for mutations of interest on non-coding genes we used all exonic and splicing mutations.

A total of 2403 mutations of interest were detected, of which 1589 were in protein-coding genes and 814 in non-coding genes (Supplementary Data 10). The average number of mutations of interest per sample was 200 (range 94–351), of which an average of 132 (range 73–222) were in protein-coding genes, and 78 were in non-coding genes (range 21–129). These alterations hit 1428 protein-coding and 764 non-coding genes, of which 145 and 61 were hit in 2 or more samples, respectively (Supplementary Data 10). Relative to SNVs, much fewer indels were called, with the average per sample was 15 (range 8–23), of which an average of 7 (range 2–18) were in protein-coding genes, and 8 were in non-coding genes (range 4–14). These alterations hit 74 protein-coding and 94 non-coding genes, of which 3 and 1 were hit in two or more samples, respectively (Supplementary Data 11).

Among the most recurrently mutated genes, APC was mutated in all high-purity samples and TP53 in 15 samples. Further recurrently mutated genes included *KRAS, NRAS, SOX9, TCF7L2*, and *FBXW7*. We observed mutations in *TTN* and *LRP1B* which have been described as passenger mutations, although *LRP1B* is a paralogue of *LRP1*, which is known to be involved in Wnt receptor signaling. *SOX9* was exclusively hit by frameshift insertions and deletions and always co-occurred with mutations in *HK3*, but this was not observed in the larger TCGA and Giannakis cohorts.

Correlating these recurrently mutated protein coding genes, ncRNAs and 3′-UTRs with clinical factors we found that *KRAS* was mutated exclusively in right sided colon and caecum tumors (compared to left sided sigmoid) and *TP53* was mutated only in left sided sigmoid (compared to right sided colon and caecum) (Supplementary Data 12) consistent with observations by Yaeger et al. Additionally, we found mutations in *RP11-983P16.2, POKR1, SLC26A10* affect females more than males (p 0.0455, *χ*^2^-test), 3′-UTRs mutations of *CBLB, IFI44L, MMP16, RNF217* affect females more than males (p 0.0455, *χ*^2^-test), and 3′-UTR mutations of *XKR4* were found 3 of 3 patients who did not undergo neoadjuvant therapy (compared to 0 of 3 who did).

The mean inter-mutation distance across the genome was between 10,000 and 1,000,000 bp and we did not observe recurrent regions of kataegis in our patients. Some of these individual kataegis loci were in close proximity to genes *PEAK1, ADAP2, SUFU*, and *SGK3*, with *SUFU* being metastasis-specific and *SGK3* tumor-specific (Supplementary Figure 10, 11). However, several recurrent regions of increased mutation density were seen in both tumor and metastases, most prominently on chromosomes 5 and 13 which may be due to a gain of partially methylated domains^62^ (Supplementary Figure 10, Supplementary Figure 11).

### Sample classification

The samples in our series were all deemed to be micro-satellite stable; they did not harbor mutations on DNA mismatch repair genes *MLH1, MLH3, MSH2, MSH3, MSH6, PMS2* suggesting that they were not micro-satellite instable/hypermutators, nor did they harbor mutations on *POLE* suggesting that they were neither ultra-mutators.

The sample exhibiting the most mutations in our series (CRC-008, primary tumor) has 17,189 somatic SNVs, equivalent to 6.1 mutations per 10^6^ bases (assuming 2.8 Gb of mappable human genome), which is about half of this hypermutator boundary. By extension, our samples cannot be classified as ultra-mutators.

### Mutual exclusivity analysis

Mutual exclusivity analysis was initially performed on all genes that have established roles in colorectal cancer. Gene pairs were deemed to be mutually exclusive if no more than 1 sample harbored somatic SNVs for them. Using cBioPortal, we determined the significance of mutual exclusivity and co-occurrence of recurrently mutated genes in our, the TCGA, Giannakis et al., and Yaeger et al. studies, and the *ARHGEF* gene family (Supplementary Data 7). We found support to our observation of mutual exclusivity of *ARHGEF7-KRAS* (TCGA, *p*-value 0.021, Fisher test) and *SOX9-TP53* (Yaeger et al., *p*-value <0.001), *NRAS-KRAS* (Yaeger et al., *p*-value <0.001). We found *SOX9* mutations co-occurred with *HK3* mutations, and with frameshift indels in *APC* as opposed to typical stop gains, although we did not observe co-occurrence of *SOX9* and *HK3* in larger cohorts.

### Survival analysis

Survival analysis (overall and disease-free) was performed using cBioPortal on the TCGA provisional dataset using *ARHGEF7* and all *ARHGEFs* combined: *ARHGEF1, ARHGEF10, ARHGEF10L, ARHGEF11, ARHGEF12, ARHGEF15, ARHGEF16, ARHGEF17, ARHGEF18, ARHGEF19, ARHGEF2, ARHGEF25, ARHGEF26, ARHGEF3, ARHGEF33, ARHGEF34P, ARHGEF35, ARHGEF37, ARHGEF38, ARHGEF4, ARHGEF40, ARHGEF5, ARHGEF6, ARHGEF7* and *ARHGEF9* (Supplementary Figure 8).

### Structural variant calling

Structural variations (SV) were called using the SOPHIA algorithm (manuscript in preparation) using a workflow as described in Sahm et al.^63^.

Briefly, SOPHIA uses information of supplementary alignments from the alignment file as produced by bwa-mem. This indicates candidate chimeric alignments of split-reads which would be an indication of a possible underlying SV. SOPHIA uses a decision tree to consider only high-quality reads that do not fall on lowly mappable regions or consist of low-quality base calls. SOPHIA uses these reads and further filters the results by comparing them to a background control set of sequencing data derived from normal blood samples from a large background population database of 3261 patients from published TCGA studies and both published and unpublished DKFZ studies, sequenced using Illumina HiSeq 2000, 2500 (100 bp) and HiSeq X (151 bp) platforms and aligned uniformly. A SV is discarded if: it has more than 75% of read support is from low-quality reads; the second breakpoint of the SV was unmappable in the sample and in 10 or more background control samples; a SV with 2 breakpoints had one present in at least 98 control samples (3% of the control samples); both breakpoints have less than 5% read support at both positions.

In addition to the recurrently hit genes, we also found a number of topologically associated domains that were recurrently hit by SVs, including chr10:13,280,000–15,440,000 (containing *SUV39H2*), chr1:3,360,000–3,359,999 (*MUM1, GNA15, GNA11, STK11, and TCF3*), chr14:67,880,000–69,720,000 (*RAD51B*), and chr19:14,600,000–16,800,000 (*BRD4*) (Supplementary Data 13).

### Copy number aberration calling

Copy number aberrations (CNAs) were called using ACEseq^64^, which is available on github [https://github.com/eilslabs/ACEseqWorkflow]. Briefly, ACEseq (allele-specific copy number estimation from whole-genome sequencing) determines copy number states, tumor cell content, ploidy, and sex in the tumor by using read coverage and the B-allele frequency (BAF). Heterozygous germline positions (with BAF 0.33–0.77 at dbSNP version 135 SNP loci)^65^ are identified for later allele-specific copy number and loss-of-heterozygosity (LOH) analysis. Phasing is performed using impute2 on heterozygous and homozygous alternative SNP positions to improve sensitivity of detection of imbalanced and balanced regions^66^. Tumor and control read coverage is calculated for 10 kb windows with sufficient mapping quality and read density, which is then corrected for GC-content and replication timing bias using linear regression, removing coverage fluctuations associated with these biases. Genome segmentation is performed using the PSCBS package in R additionally including the previously identified SV breakpoints^67^. Small segments (<9 kb) are merged to their most similar neighboring segment. Segments are c-means clustered according to their coverage ratio and BAF. Neighboring segments are joined if they belong to the same cluster. Sample ploidy and tumor cell content are estimated by scanning different ploidy and purity combinations and selecting the ones that best described the data. As a constraint, balanced BAF segments are fitted to even-numbered copy number states but unbalanced BAF segments were additionally fitted to uneven numbers. Then the allele-specific copy number for each segment is calculated using the fitted estimated tumor cell content and ploidy.

For subsequent analysis, gains and losses were identified when they deviate more than 0.7 from the base ploidy. Annotation of genes was based on direct overlap with gene models from gencode version 19.

We observed recurrent chromosome arm-level changes, included gains 7p and q, 8q, 13q, 19q, and 20p and q, and deletions in 1p, 4q, 8p, 15q, 17p, and 18q, which have been described in the TCGA study^5^. In addition, we observed recurrent amplifications of chromosome arms 6p and q and 16p and losses in 4p, 5q, 8p (contrary to TCGA), and 18p and Y (in males). Chromosomes 8, 13, 18, and 20 were observed to have the most recurrent alterations. Six patients harbored CNAs on all of these chromosomes.

### Identification of kataegis loci

Kataegis loci were classified as clusters of a minimum of 5 mutations within a 10 kb region. Annotation of genes to kataegis loci was done using bedtools using the gencode version 19 gene models. A kataegis locus was determined to be proximal to a gene if it was within 10 kb of it.

### Supervised mutational signatures analysis

Supervised mutational signatures analysis was performed using the R package YAPSA [https://rdrr.io/bioc/YAPSA/]. The linear combination decomposition of the mutational catalog with known and predefined COSMIC signatures^68^ was computed by non-negative least squares (NNLS) as described in Giessler et al.^69^. The mutational signature analysis was applied to the mutational catalogs for SNVs of the 8 high-purity paired tumors and metastasis samples individually and tumor-specific and metastasis-specific mutations per patient. A signature-specific cutoff was applied and cohort level analysis was used for detecting signatures.

### Ingenuity pathway analysis

All genes hit by non-synonymous, including stop gain SNVs and indels were imported into the core analysis pipeline of the Ingenuity Pathway Analysis tool. Genes that were hit multiple times were included as an individual entry. SNVs and indels were combined together and exonic mutations in primary tumors (822 genes), metastasis (913 genes), primary tumor 3′-UTRs (770 genes) and metastasis 3′-UTRs (809 genes) were analyzed individually. Core analysis was performed with the default settings and the most significant pathways were selected after removal of those unrelated to cancer, GI disease or colorectal physiology.

### Annotation enrichment analysis with DAVID

All genes hit by metastasis-specific mutations and indels that were mutated at least twice as much in metastasis samples compared to tumors were imported into functional annotation clustering tool of DAVID. The homo sapiens background, medium stringency and Benjamini-Hochberg correction was applied to the hypergeometric test.

### In silico evaluation of miRNA binding to mutated 3′-UTRs

All predictions were made with the RNA22 interactive software [https://cm.jefferson.edu/rna22/Interactive/] using all known miRNA (miR) sequences from miRBase (Release 21) and the corresponding wild-type or mutated sequences as input. Default settings were used with sensitivity at 63%, specificity at 61%, seed size of 7 with a maximum of one unpaired base. The minimum number of paired-up bases in the heteroduplex was 12, the maximum folding energy for the heteroduplex (Kcal/mol) was −515 and no limit was given on the number of potential GU wobbles in the seed region. Gain- or loss-of-potential miRNA binding was evaluated by positive results in the presence or absence of a given mutation.

## Electronic supplementary material

Supplementary Information

Description of Additional Supplementary Files

Supplementary Data 1

Supplementary Data 2

Supplementary Data 3

Supplementary Data 4

Supplementary Data 5

Supplementary Data 6

Supplementary Data 7

Supplementary Data 8

Supplementary Data 9

Supplementary Data 10

Supplementary Data 11

Supplementary Data 12

Supplementary Data 13

## Data Availability

The whole-genome sequencing data have been deposited at the European Genome-phenome Archive (EGA). The EGA Study Accession ID is EGAS00001002717. All the other data supporting the findings of this study are available within the article and its supplementary information files and from the corresponding author upon reasonable request.
