# Supplementary material for: Whole genome sequencing puts forward hypotheses on metastasis evolution and therapy in colorectal cancer
Source: Nat Commun. 2018 Nov 14;9:4782. doi: 10.1038/s41467-018-07041-z (PMC6235880; doi:10.1038/s41467-018-07041-z)
Supplement: Supplementary file 1 — Supplementary Information [file 41467_2018_7041_MOESM1_ESM.pdf]

Supplementary information

[**Article**: Whole genome sequencing reveals novel hypotheses on metastasis evolution and therapy in colorectal cancer]

Ishaque et al.

Supplementary Figures 1 - 11

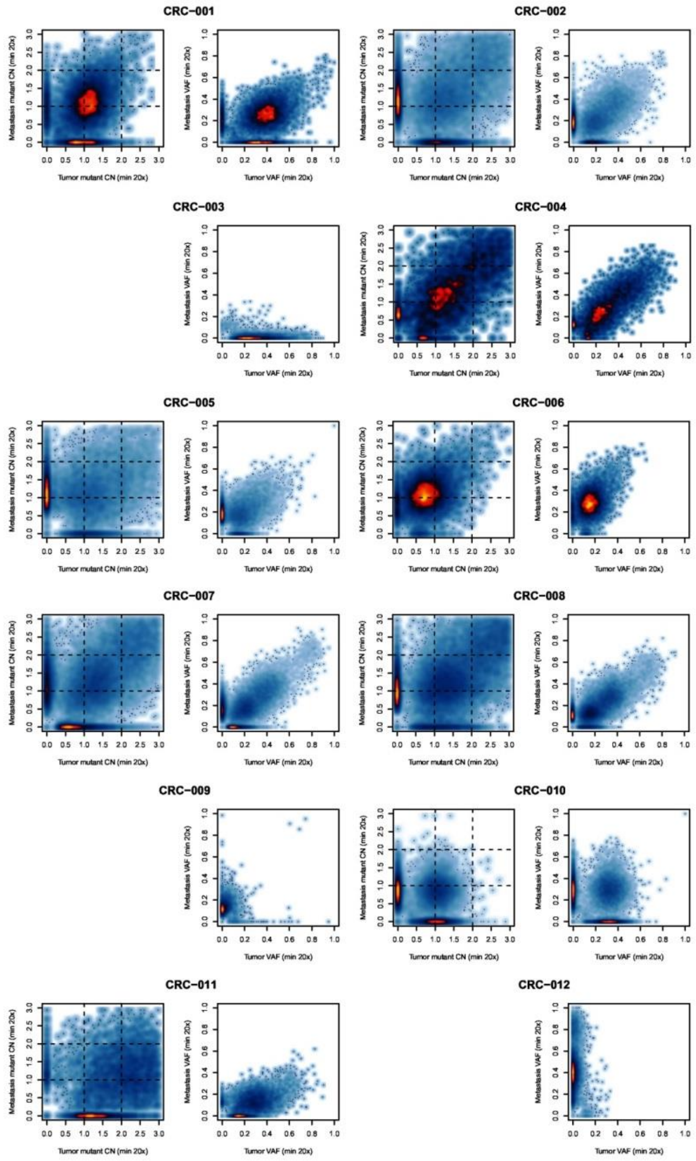

Supplementary Figure 1. <u>Small mutation copy number and variant allele fraction comparison of tumor and metastasis</u>. Heatmap representation of density of mutational copy number (left) and variant allele fraction (VAF) (right) of somatic SNVs and indels in tumor (x-axis) and metastasis (y-axis) for each sample; only variants with at least 20x coverage are shown. Samples with missing mutation copy number plots had low tumor cell content for either/both the tumor and metastasis samples. The heatmap scale is white, blue, red, yellow from low to high. Sample exhibiting sub-clonality of tumor (cloud of points close to x-axis) or metastasis (cloud of points close to the y-axis) compared to the shared truncal origin (cloud of points in the body of the heatmap) can be seen, for example in sample CRC-004.

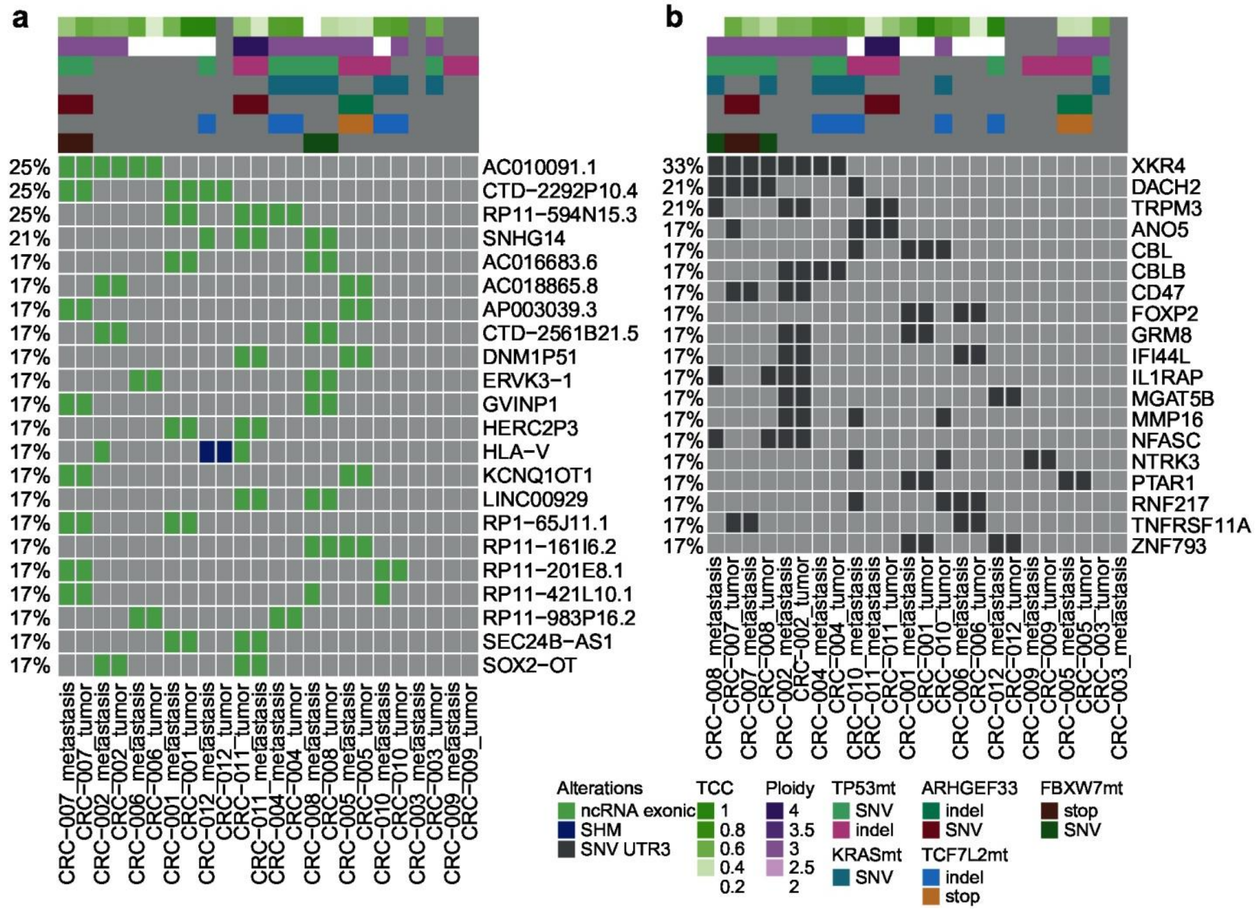

Supplementary figure 2: Recurrent non coding mutations. Oncoprint representation of recurrently mutated (minimum of 4 samples, 17%) non-coding RNA (A) and 3' UTR regions (B). The color of the box represents the mutation type. The top heatmap annotations shows ploidy, estimated TCC and mutational status for *TP53*, *KRAS*, *ARHGEF33*, *TCF7L2*, and *FBXW7*.

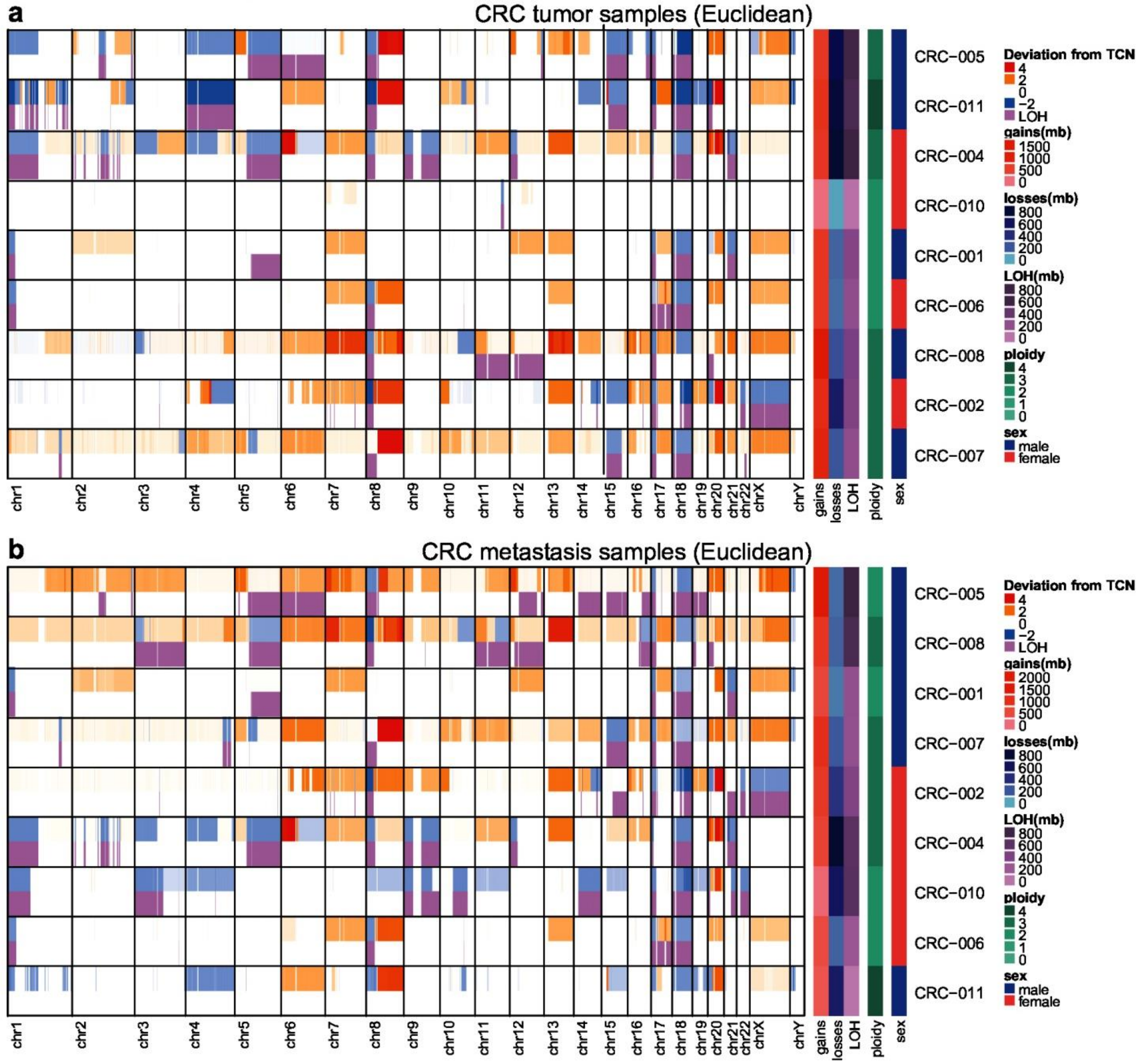

Supplementary figure 3: Copy number profiles of tumor and metastasis samples. Heatmap like representation of copy number profiles for tumors (A) and metastases (B), where samples (rows) are clustered (Euclidean) by similarity of chromosome copy number profiles (columns). Each sample is represented by 2 rows, the top row indicating copy number gains (orange) and losses (blue), and the second row indicating loss of heterozygosity (LOH) (purple). The row annotations, from left to right: amount of chromatin (Mb) that have copy number gains, losses, LOHs, predicted ploidy, and sex. Samples with purity lower than 30% were not used (therefore 9 of 12 were used for both tumors and metastasis, table 1).

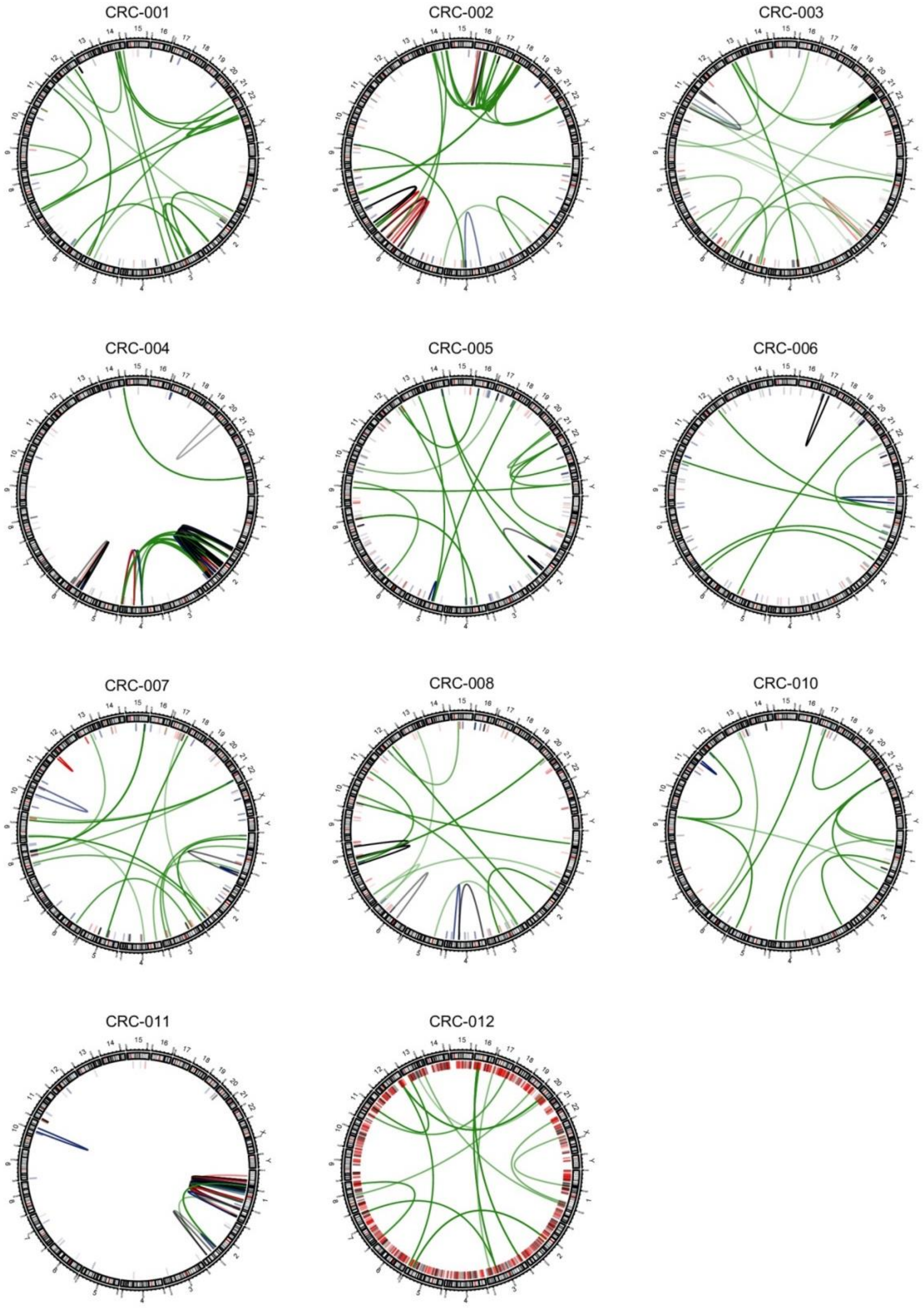

Supplementary figure 4: Structural variations in tumor samples. Circular representations of somatic structural variations within tumor samples. The outer ring shows chromosome number and chromosome cytobands. Arcs represent translocations (green), inversion (black), deletions (blue) and amplifications (red).

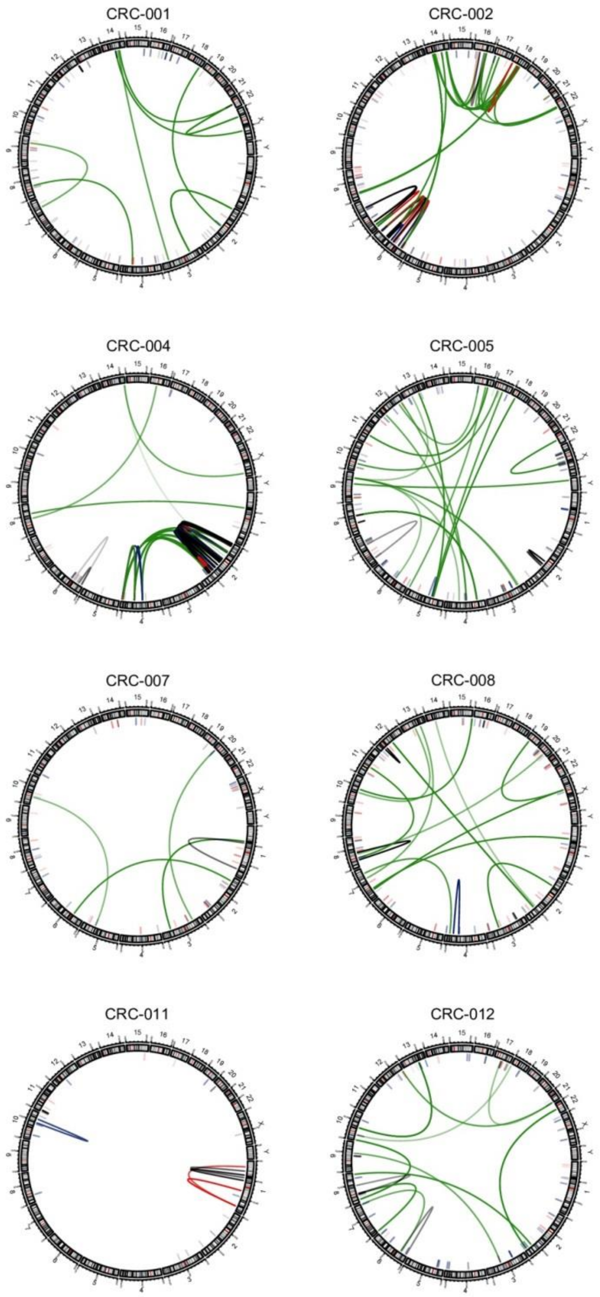

Supplementary figure 5: Structural variations in metastasis samples. Circular representations of somatic structural variations within metastasis samples. The outer ring shows chromosome number and chromosome cytobands. Arcs represent translocations (green), inversion (black), deletions (blue) and amplifications (red).

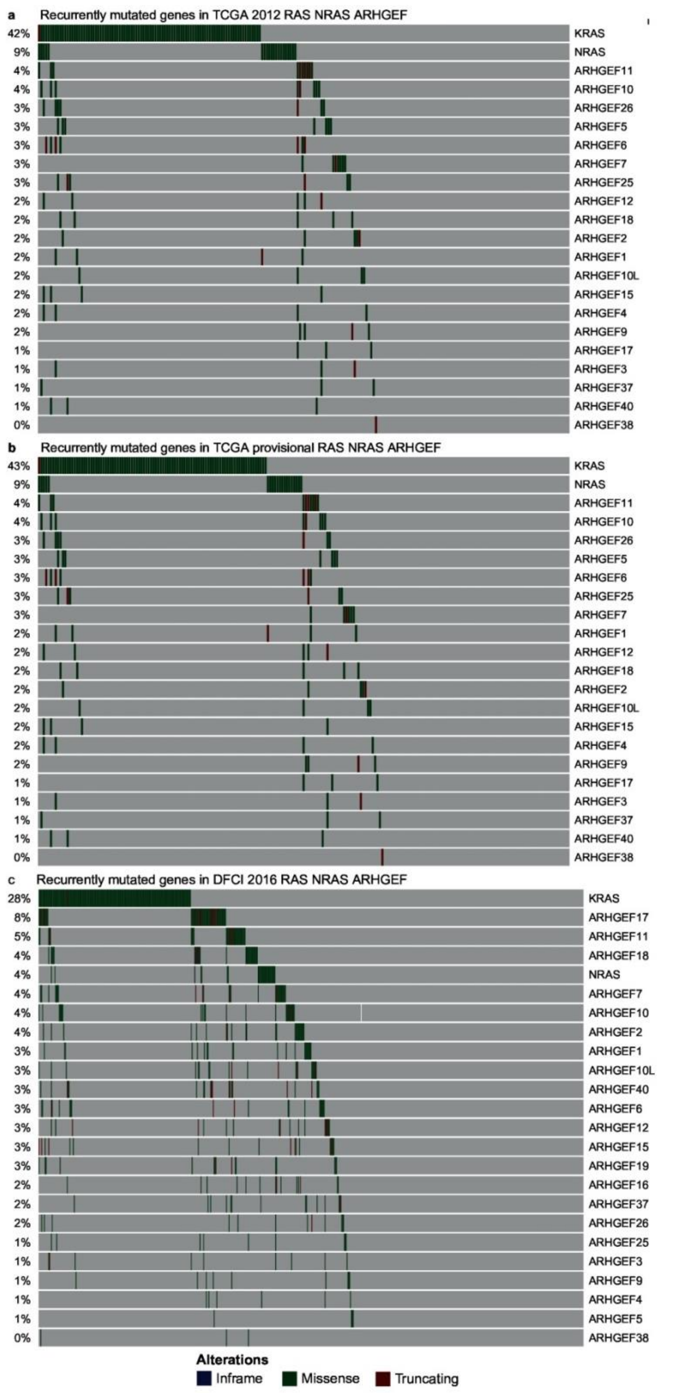

Supplementary figure 6: <u>Recurrent somatic small mutations on *KRAS*, *NRAS* and *ARHGEF* genes in larger cohort studies.</u> Oncoprint representation of recurrently mutated RAS and *ARHGEF* genes. Mutations were taken from cBioPortal for the (a) TCGA 2012, (b) TCGA provision, (c) DFCI/Giannakis et al., (d) MSK-CC/Yaeger et al. all samples and (e) MSK-CC/Yaeger et al. patients with matched tumor-metastasis. The alterations represented in the oncoprint are in-frame (blue), missense (green) and truncating (red) mutations.

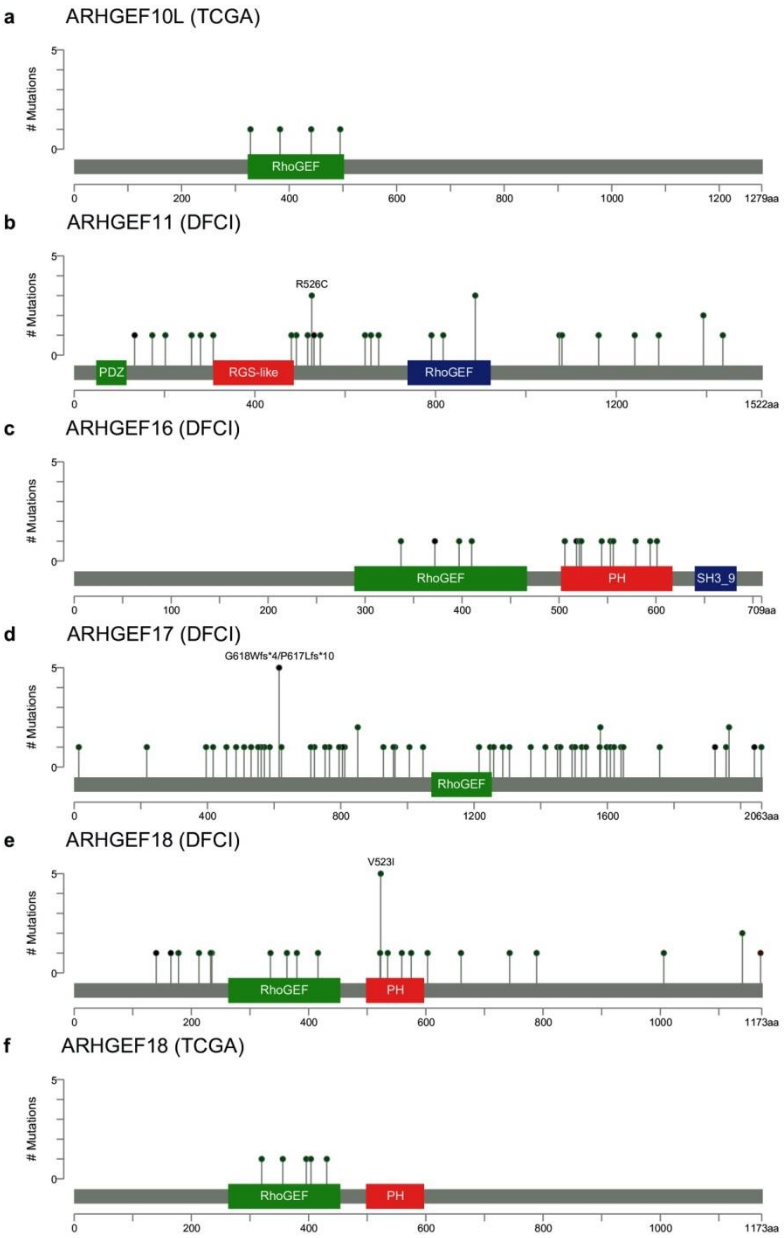

Supplementary figure 7: <u>Lollipop plots of *ARHGEF* genes from other studies.</u> Lollipop plots of *ARHGEF* genes with recurrent and/or clusters of mutations implicating the importance of the RhoGEF and PH domains. For each panel, the y-axis shows the number of samples with a given mutation, and the x-axis shows different positions along the coding region of the gene. The genes shown are (a) *ARHGEF10L* from the DFCI dataset, (b) *ARHGEF11* from DFCI, (c) *ARHGEF16* from DFCI, (d) *ARHGEF17* from DFCI, (e) *ARHGEF18* from DFCI, (f) *ARHGEF18* from the TCGA dataset.

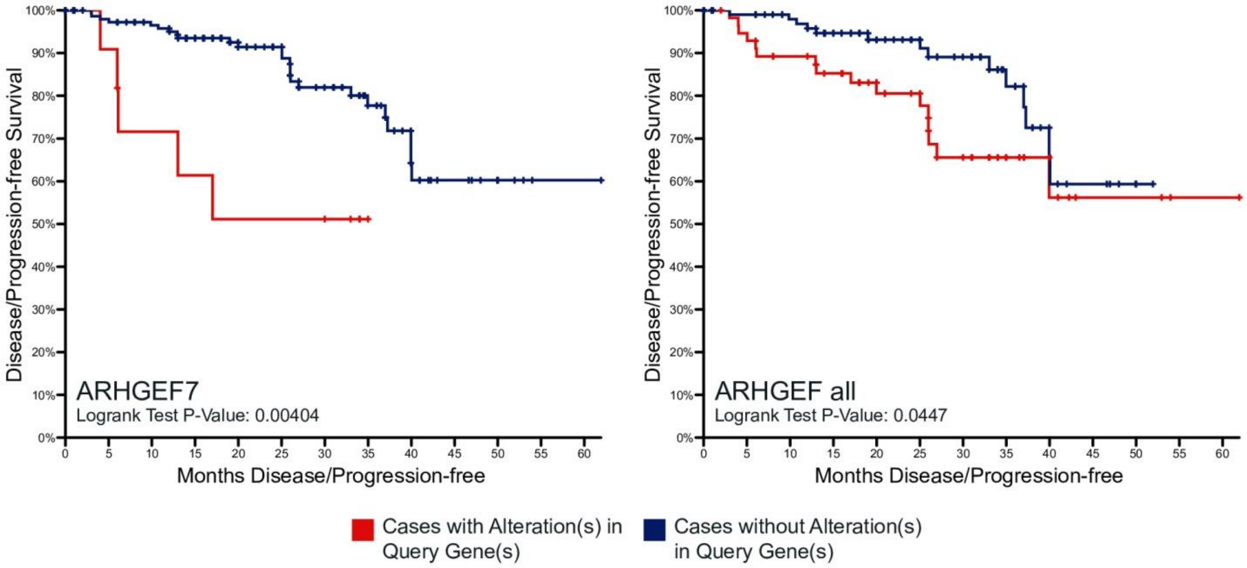

Supplementary figure 8: Disease free survival Kaplan Meier curves for *ARHGEF7* and all *ARHGEF* genes. Kaplan Meier curves for different genes/gene-sets described within the study. The red curve denotes survival of patients harboring the mutation, and blue denotes survival of patients that do not. Significance is reported as log rank test *p*-value. Patient data is from the TCGA provisional study from cBioPortal.

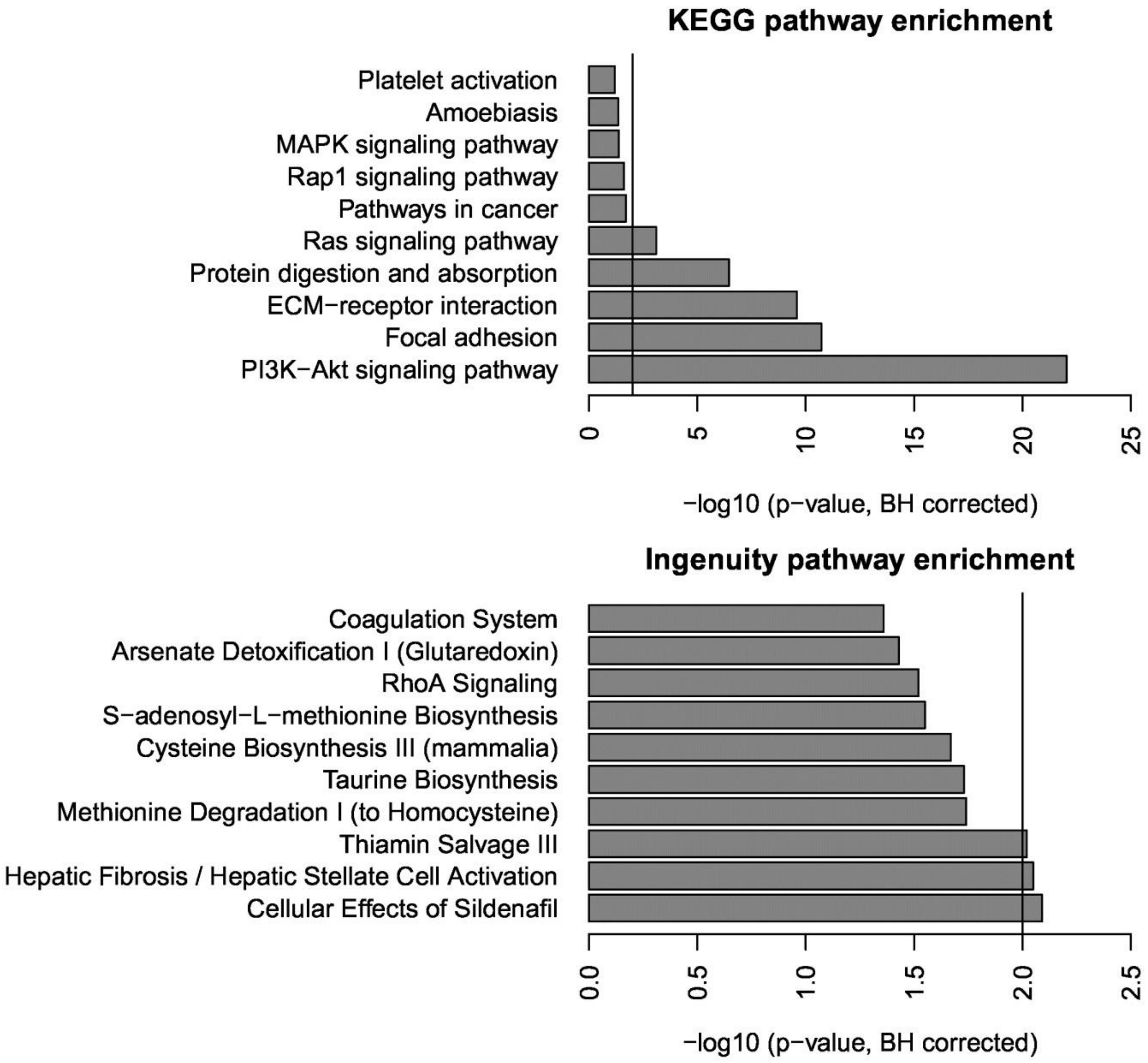

Supplementary figure 9: Pathway enrichment of metastasis specific mutations. Bar chart representation of the top 10 enriched terms from KEGG (A) and Ingenuity (B) pathway analysis of metastasis specific mutations. The terms are named in the y-axis and the –log10 (Benjamini-Hochberg correct *p*-value) are shown in the x-axis. A vertical line is depicted for x=2 which equates to a corrected *p*-value of 0.05.

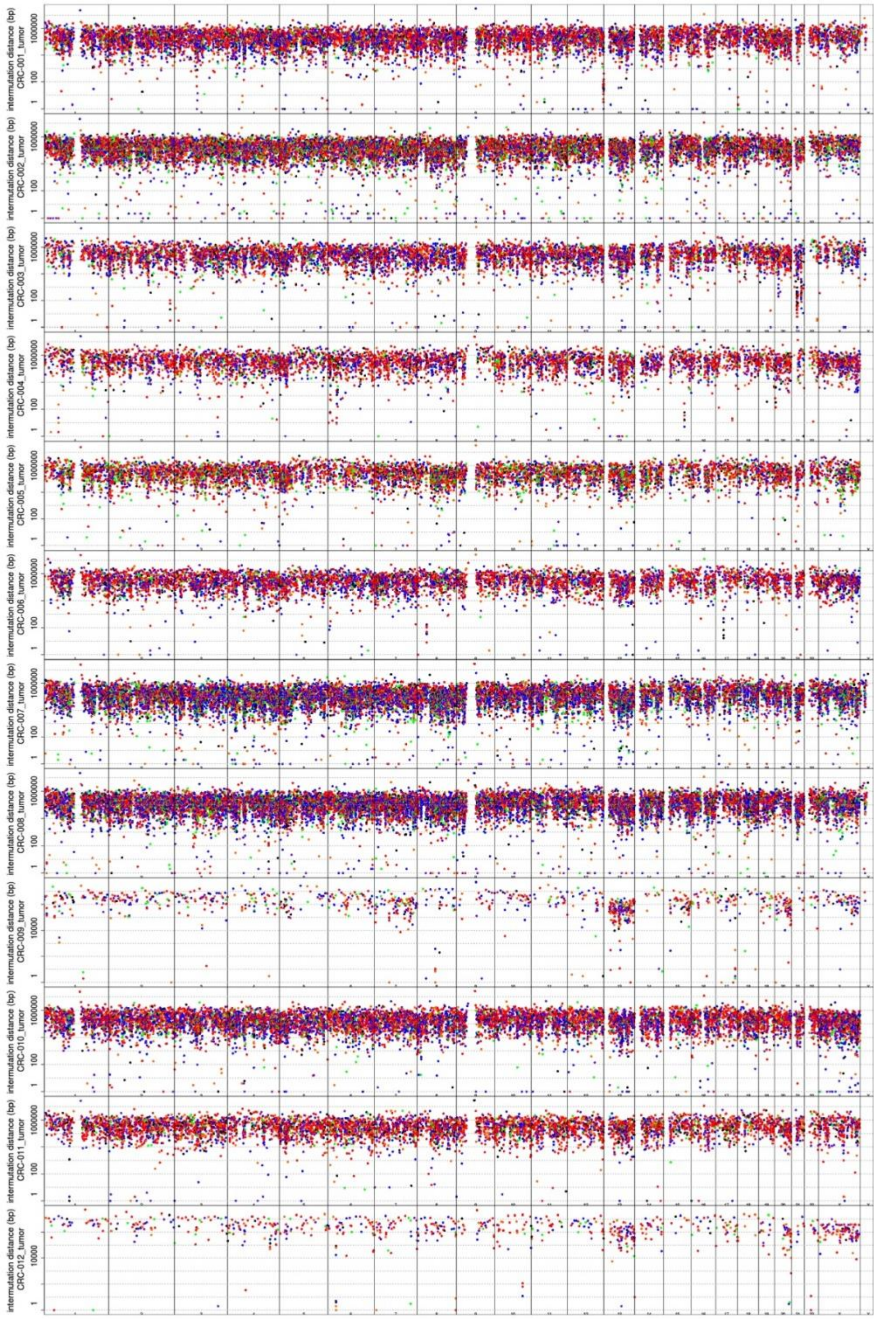

Supplementary figure 10: Intermutational distance plot of somatic SNVs in tumor samples. Plots showing the Intermutational distance (bp) between somatic SNVs (x-axis) throughout different chromosomes (y-axis). The dots represent different types of mutations: C>A (blue), C>G (black), C>T (red), T>A (purple), T>C (orange), and T>G (green). Examples of kataegis "rainfalls" can be seen for sample CRC-001 at the end of chromosome 12. Increased mutational density can be seen for chromosome 13 for a number of samples.

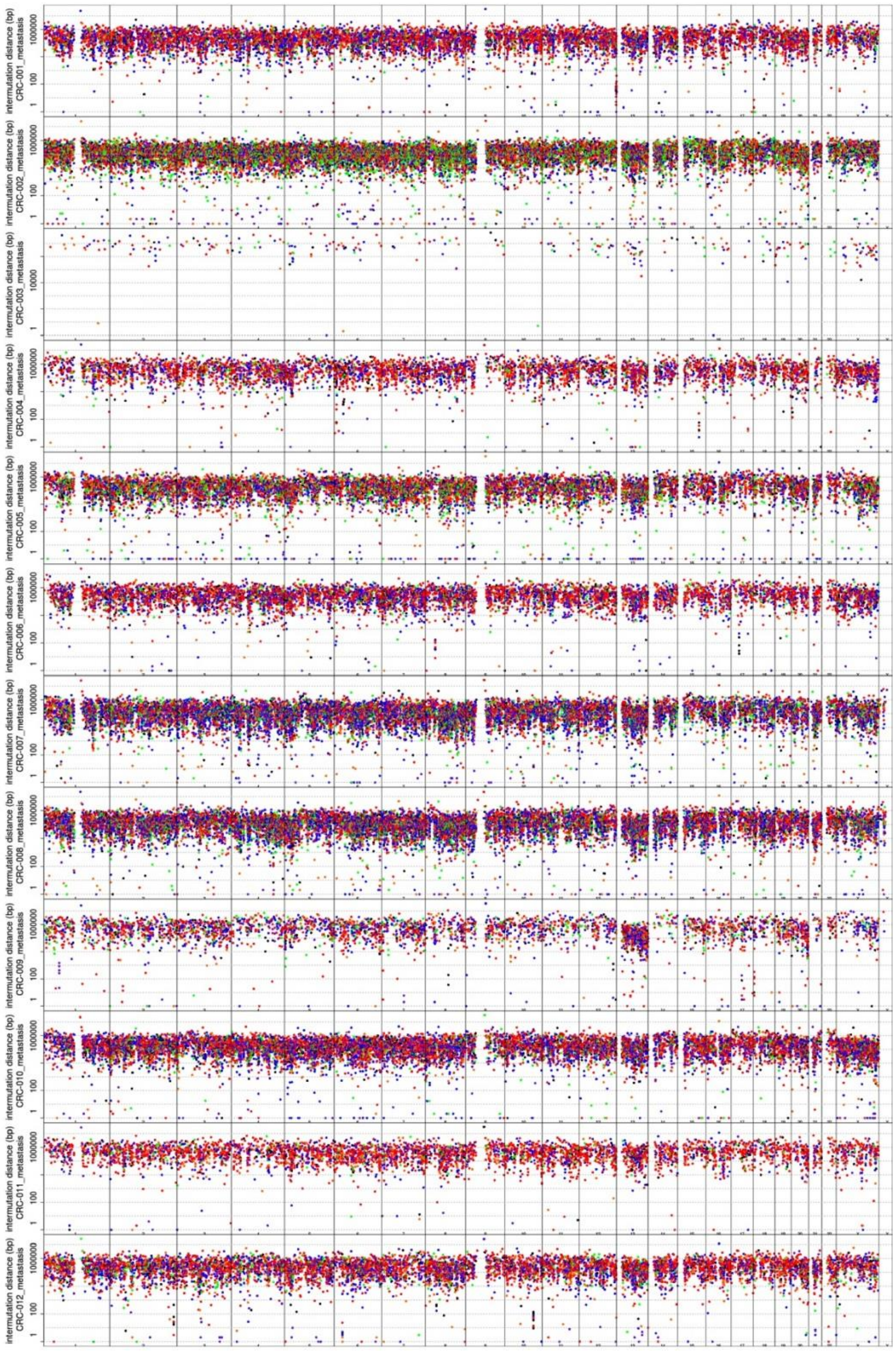

Supplementary figure 11: Intermutational distance plot of somatic SNVs in metastasis samples . Plots showing the Intermutational distance (bp) between somatic SNVs (x-axis) throughout different chromosomes (y-axis). The dots represent different types of mutations: C>A (blue), C>G (black), C>T (red), T>A (purple), T>C (orange), and T>G (green). Examples of kataegis “rainfalls” can be seen for sample CRC-001 at the end of chromosome 12. Increased mutational density can be seen for chromosome 13 for a number of samples.
