# Supplementary material for: Whole genome sequencing puts forward hypotheses on metastasis evolution and therapy in colorectal cancer
Source: Nat Commun. 2018 Nov 14;9:4782. doi: 10.1038/s41467-018-07041-z (PMC6235880; doi:10.1038/s41467-018-07041-z)
Supplement: Supplementary file 2 — Description of Additional Supplementary Files [file 41467_2018_7041_MOESM2_ESM.pdf]

Description of Additional Supplementary Files

File Name: Supplementary Data 1
Description: Sequence alignment statistics. Table displaying the alignment statistics for each sample, predicted insertsize and percentage of read pairs mapping to different chromosomes (Diff Chrom %).

File Name: Supplementary Data 2
Description: Small mutations shared between tumor and metastasis samples. The number and percentage of small mutations for each patient's metastasis, tumor, shared, tumorspecific, metastasis specific mutations. Various summaries are presented below, for all samples, for all samples where both tumor and metastasis have above 30% tumor cell content (TCC), for samples where both tumor and metastasis samples have above 30% TCC and the TCC varies less than 10% or 15% between tumor and metastasis samples.

File Name: Supplementary Data 3
Description: Recurrent mutations of non-coding genes. Table of recurrently mutated non-coding genes, ordered by the recurrence. The number and names of tumor and metastasis samples are listed.

File Name: Supplementary Data 4
Description: In-silico predictions of altered miRNA binding caused by 3' UTR mutations. Two representative examples of distortions in miRNA binding as a result of SNVs/indels. In the first example, a C to A mutation causes loss of regulation of the designated miRNAs which all bind to the wild-type sequence. Changing the C to A shifts the folding energy of the heteroduplex making binding unfavorable. As a result, the mutated sequence can potentially not be regulated by the designated miRNAs. In the second example, two consecutive sequential mutations cause a change from the wild-type AA to TC. As a result, the designated miRNAs whose binding/folding energy was unfavorable now become significantly enhanced. In both examples, affected nucleotides are highlighted in a red box. The upper sequence is the 3'UTR sequence in 5'-3' orientation and the bottom sequence is the designated miRNA in 3' to 5' orientation. Connecting lines signify complementary Watson-Crick bonding and two dots represent GU wobbles. All predictions were made with the RNA22 interactive software (https://cm.jefferson.edu/rna22/Interactive/) using miRNA sequences from miRbase and the corresponding wild-type or mutated sequences as input. Default settings were used with sensitivity at 63%, specificity at 61%, and seed size of 7 with a maximum of one unpaired base. The minimum number of paired-up bases in the heteroduplex was 12, the maximum folding energy for the heteroduplex (Kcal/mol) was -12 and no limit was given on the number of potential GU wobbles in the seed region.

File Name: Supplementary Data 5
Description: Genes recurrently targeted by structural variants. Table of genes recurrently targeted by structural (SVs) listed by frequency. Genes were either directly hit by an SV, or were the closest gene to an SV event. Counts for combined, tumor and metastasis samples are shown.

File Name: Supplementary Data 6
Description: Mutual exclusivity analysis. Significance and trends are from cBioPortal for the TCGA (2012 and provision), Giannakis et al. (DFCI) and Yaeger et al. (MSK-CC) studies, and the ARHGEF gene family. Further filtering for the lowest p values, and overall trend was done (based on frequency).

File Name: Supplementary Data 7
Description: DAVID functional annotation cluster enrichment of metastasis specific mutations. Table of DAVID functional annotation clustering of metastasis specific mutations, where genes are present in the metastasis sample but not the tumor and where the number of samples harboring mutations on a gene in the metastasis set is >=2 x as much as the tumors. Genes and program parameters are listed.

File Name: Supplementary Data 8
Description: DKFZ NCT-MASTER targetable cancer lesions. List of targetable 831 genes harboring mutations in this study. Mutations types are SNVs (SNV), indels (INDEL), large copy number aberrations (BIG_CNV), focal copy number aberrations (FOCAL_CNV), structural variants (SV) and loss of heterozygosity (LOH).

File Name: Supplementary Data 9
Description: Targetable genes with 3’ UTR mutations. A table of patient’s samples and mutations in 3’ UTRs of genes that can be targeted by a known drug of antibody.

File Name: Supplementary Data 10
Description: Genes affected by somatic SNVs. Tables for different gene classes being affected for each patient in their tumor sample, metastasis sample and combined.

File Name: Supplementary Data 11
Description: Genes affected by somatic Indels. Tables for different gene classes being affected for each patient in their tumor sample, metastasis sample and combined.

File Name: Supplementary Data 12
Description: Correlation of recurrently mutated coding genes, ncRNA and 3'UTRs with clinical factors. Significance was calculated by Chi Square test, uncorrected, using an expectation model based on sample size.

File Name: Supplementary Data 13
Description: TADs recurrently targeted by structural variants. Table of TADs recurrently targeted by structural (SVs) and genes therein listed by frequency. TADs were directly hit by an SV. Counts for combined, tumor and metastasis samples are shown.
